# Brain Metastasis from *EGFR*‐Mutated Non‐Small Cell Lung Cancer: Secretion of IL11 from Astrocytes Up‐Regulates PDL1 and Promotes Immune Escape

**DOI:** 10.1002/advs.202306348

**Published:** 2024-05-02

**Authors:** Mengyi Tang, Mingxin Xu, Jian Wang, Ye Liu, Kun Liang, Yinuo Jin, Wenzhe Duan, Shengkai Xia, Guohui Li, Huiying Chu, Wenwen Liu, Qi Wang

**Affiliations:** ^1^ the Second Affiliated Hospital of Dalian Medical University 467 Zhongshan Road Dalian 116027 China; ^2^ Laboratory of Molecular Modeling and Design State Key Laboratory of Molecular Reaction Dynamics Dalian Institute of Chemical Physics Chinese Academy of Science 457 Zhongshan Road Dalian 116023 China; ^3^ Cancer Translational Medicine Research Center The Second Hospital, Dalian Medical University 467 Zhongshan Road Dalian 116027 China

**Keywords:** astrocytes, brain metastasis, epidermal growth factor receptor, immune escape, interleukin‐11

## Abstract

Patients who have non‐small cell lung cancer (NSCLC) with epidermal growth factor receptor (*EGFR*) mutations are more prone to brain metastasis (BM) and poor prognosis. Previous studies showed that the tumor microenvironment of BM in these patients is immunosuppressed, as indicated by reduced T‐cell abundance and activity, although the mechanism of this immunosuppression requires further study. This study shows that reactive astrocytes play a critical role in promoting the immune escape of BM from *EGFR*‐mutated NSCLC by increasing the apoptosis of CD8^+^ T lymphocytes. The increased secretion of interleukin 11(IL11) by astrocytes promotes the expression of PDL1 in BM, and this is responsible for the increased apoptosis of T lymphocytes. IL11 functions as a ligand of EGFR, and this binding activates EGFR and downstream signaling to increase the expression of PDL1, culminating in the immune escape of tumor cells. IL11 also promotes immune escape by binding to its intrinsic receptor (IL11Rα/glycoprotein 130 [gp130]). Additional in vivo studies show that the targeted inhibition of gp130 and EGFR suppresses the growth of BM and prolongs the survival time of mice. These results suggest a novel therapeutic strategy for treatment of NSCLC patients with *EGFR* mutations.

## Graphical abstract

Increased secretion of IL11 by reactive astrocytes, which binds to EGFR and gp130 on tumor cells and activates the downstream AKT signaling, promotes PDL1 expression in *EGFR*‐mutated NSCLC BM cells and increases apoptosis of T cells in the brain TME, providing a novel therapeutic approach that combined targeting gp130 with EGFR‐TKIs for the treatment of patients with *EGFR*‐mutant NSCLC BM.

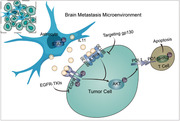

## 1 Introduction

Lung cancer is the major cause of cancer deaths worldwide, and about one‐quarter of patients who initially present with stage IV non‐small cell lung cancer (NSCLC) also have brain metastasis (BM).^[^ ^1^ ^]^ Because of the unique anatomy and physiological function of the brain, there are limited treatment options for these patients.^[^ ^2^ ^]^ Although radiotherapy is the standard treatment for BM, the median overall survival time of these patients is only 4 to 8 months.^[^ ^3^ ^]^

Epidermal growth factor receptor (*EGFR*) is an oncogene, and mutations that lead to its activation promote the pathogenesis of NSCLC. At the molecular level, mutations of *EGFR* promote the spontaneous dimerization and activation of this protein, and this contributes to the onset and progression of NSCLC.^[^ ^4^ ^]^ NSCLC patients with certain *EGFR* mutations also have an increased risk of BM.^[^ ^5^, ^6^, ^7^ ^]^ Osimertinib, an EGFR tyrosine kinase inhibitor (EGFR‐TKI), provides significant benefits to NSCLC patients with *EGFR* mutations, including those with BM, but these patients inevitably experience cancer progression because of the development of drug resistance.^[^ ^8^ ^]^

Recent studies of NSCLC found that compared with the primary lung tumor, the tumor microenvironment (TME) of BM is significantly immunosuppressed, as indicated by reduced T‐cell abundance and activity.^[^ ^9^, ^10^, ^11^ ^]^ This suggests that a defect in the immunoediting process is critical for the formation of secondary metastases in the cerebrum. The immune checkpoint programmed cell death protein 1 (PD1) and its ligand (PDL1) are crucial factors that function in the immune escape stage of immunoediting. PDL1 is on the surface of tumor cells, and it binds with its receptor (PD1) on the surface of T cells, thereby inhibiting the cytotoxic effects of T cells.^[^ ^12^ ^]^ Anti‐tumor responses mediated by immune checkpoint inhibitors (ICIs) rely on the expression of PDL1 in tumors and the infiltration of T cells capable of recognizing and killing tumor cells.^[^ ^13^ ^]^ However, the efficacy of these treatments against NSCLC with *EGFR* mutations remains limited.^[^ ^14^, ^15^ ^]^ Immune cells such as CD8^+^ T cells are associated with prolonged survival of cancer patients and with increased efficacy of immunotherapy.^[^ ^16^ ^]^ A lack of T cells in tumors can lead to resistance to immunotherapy.^[^ ^17^ ^]^ Studies of the mechanism of decreased T‐cell abundance and activity in the TME of BM may help to identify better biomarkers for prediction of clinical response and to develop new immunotherapy targets for more effective combination therapies that can overcome immune resistance.

Although activation of EGFR can increase the expression of PDL1,^[^ ^18^, ^19^ ^]^ the specific underlying mechanism of this response in the brain TME is uncertain. The TME of BM is characterized by the accumulation of reactive astrocytes and formation of carcinoma‐astrocyte gap junctions, and these changes are required for brain colonization by metastatic cells.^[^ ^20^ ^]^ Astrocytes are the most abundant glial cells in the brain microenvironment, and they undergo a transition from the naive state to a metastasis‐promoting reactive state in patients with NSCLC.^[^ ^21^ ^]^ There is evidence that reactive astrocytes assist in the tumor immune escape process,^[^ ^22^ ^]^ but the specific mechanism is unknown.

In the present study, we investigated the stimulatory effects and mechanism of reactive astrocytes in mediating immune escape in BM from *EGFR*‐mutated NSCLC. We focused on the increased secretion of IL11 by reactive astrocytes and activation of EGFR and the IL11Rα/gp130 signaling pathway, responses that lead to increased expression of PDL1 in BM cells and increased apoptosis of T cells in the brain TME. We also examined the ability of IL11 to bind and activate EGFR. Our broader purpose was to provide evidence for use of a novel therapeutic approach for the treatment of BM in patients with *EGFR*‐mutated NSCLC.

## 2 Results

### 2.1 BM from *EGFR*‐Mutated NSCLC has a Stronger Immunosuppressive Phenotype

**Figure 1:**
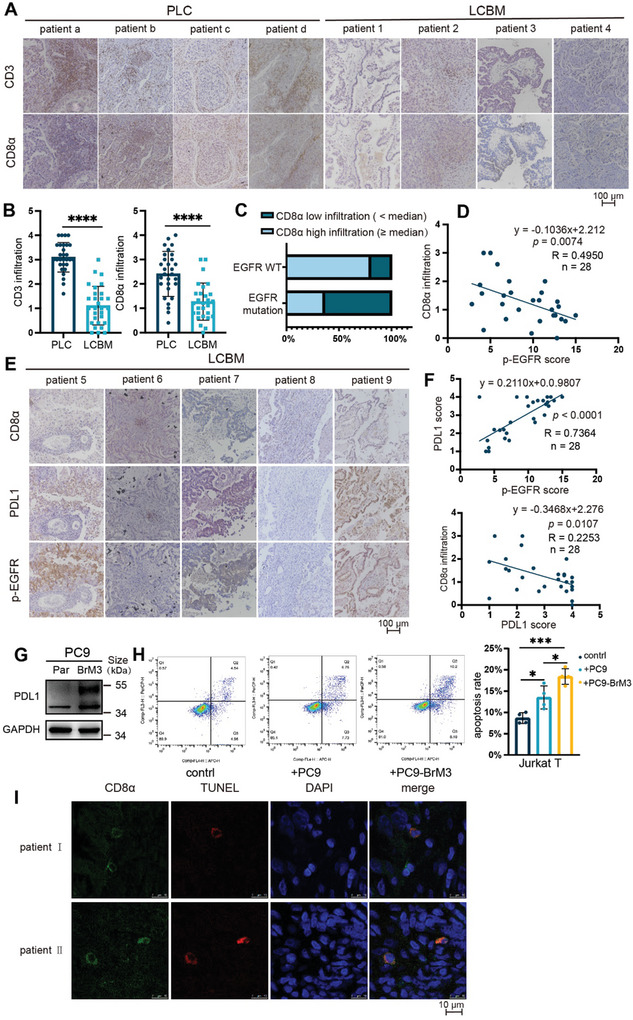
BM from *EGFR*‐mutated NSCLC has a stronger immunosuppressive phenotype. A,B) Immunohistochemistry of CD3 and CD8α in cancer tissues of patients with primary lung cancer (PLC) or LCBM (scale bar: 100 µm), with quantitation of these results for CD3 (LCBM: *n* = 29; PLC: *n* = 29) and CD8α (LCBM: *n* = 28; PLC: *n* = 29), and comparisons using an unpaired, two‐tailed *t*‐test. C) Percentage of samples with high infiltration or low infiltration of CD8α among LCBM samples with and without *EGFR* mutations, determined by Sanger sequencing (mutation: *n* = 11; WT: *n* = 5). D) Relationship of CD8α infiltration score with p‐EGFR score in LCBM samples (*n* = 28, simple linear regression). E,F) Immunohistochemistry of CD8α, PDL1, and p‐EGFR in LCBM patients (scale bar: 100 µm), and relationship of these staining scores (*n* = 28, simple linear regression). G) Western blotting of PDL1 in PC9 parental cells (left) and brain metastatic derivatives (right). H) Flow cytometry results of Jurkat T cells (left) that were co‐cultured alone, with PC9 cells, or with PC9‐BrM3 cells for 24 h, and then harvested for analysis of apoptosis, indicated by dots in the right boxes (Q2+Q3). Quantitation of the results (right), with comparisons using an unpaired two‐tailed *t*‐test. I) CD8α, TUNEL, and DAPI staining of tumor sections from two *EGFR*‐mutated LCBM patients (scale bar: 10 µm). Western blotting and flow cytometry results show representative samples from three or more replicates. *(*p* < 0.05), **(*p* < 0.01), ***(*p* < 0.001), and ****(*p* < 0.0001).

T lymphocytes, especially the CD8^+^ subset, function as the main “executioners” in the anti‐tumor immune response.^[^ ^23^ ^]^ We therefore assessed infiltration by T cells and CD8^+^ T lymphocytes by performing a retrospective analysis of CD3 and CD8α expression in the cancer tissues of patients from the Second Hospital of Dalian Medical University who had lung cancer brain metastases (LCBM) or primary lung cancer (PLC). The results showed there was significantly reduced infiltration of T‐cells and CD8^+^ T lymphocytes in patients with LCBM (**Figure** **1A,B**; LCBM: *n* = 29, PLC: *n* = 29 for CD3 staining; LCBM: *n* = 28, PLC: *n* = 29 for CD8α staining). Analysis of the clinical and demographic characteristics of the two groups (Tables S1 and S2, Supporting Information) showed they had no significant differences in age, gender, or histology (*p* > 0.05). We then examined whether T cell infiltration in LCBM was related to *EGFR* mutation status by use of Sanger sequencing and staining for phosphorylated EGFR (p‐EGFR). The results show decreased infiltration by CD8^+^ T lymphocytes in BM when the cells had *EGFR* mutations (Figure 1C) and that the extent of CD8^+^ T lymphocyte infiltration was negatively correlated with the level of p‐EGFR (Figure 1D,E; LCBM, *n* = 28). These clinical results indicate that activation of EGFR decreased the abundance T lymphocytes in LCBM.

PDL1 is expressed on tumor cells and plays an important role in inhibiting the cytotoxic effects of T cells.^[^ ^12^, ^24^ ^]^ We therefore analyzed the expression of PDL1 by staining clinical tissue samples of patients with LCBM. The results showed that the expression of PDL1 had a positive correlation with p‐EGFR and a negative correlation with infiltration by CD8^+^ T lymphocytes (Figure 1E,F; LCBM, *n* = 28). We also measured the expression of PDL1 in two previously established cell lines of BM: H2030‐BrM (*EGFR* wild type [WT]) and PC9‐BrM3 (*EGFR* ^Δexon19^ mutation).^[^ ^25^ ^]^ The western blotting results indicated that PDL1 was significantly upregulated in the PC9‐BrM3 cells relative to the parental PC9 cells (Figure 1G; Figure S1A, Supporting Information). However, this effect did not occur in H2030 cells (Figure S1B, Supporting Information).

We then co‐cultured different tumor cells with Jurkat T cells, and used flow cytometry to measure the apoptosis of the Jurkat T cells. The results showed that PC9‐BrM3 cells increased the apoptosis of Jurkat T cells more than the parental PC9 cells (Figure 1H). We also confirmed the apoptosis of CD8^+^ T lymphocyte by analysis of CD8α, TUNEL, and DAPI staining of tumor sections from LCBM patients with *EGFR* mutation (Figure 1I). Collectively, these results demonstrate that LCBM with mutated *EGFR* had a stronger immune escape function, and this was related to the upregulation of PDL1 and the promotion of apoptosis in CD8^+^ T lymphocytes.

### 2.2 Reactive Astrocytes in the Brain TME Facilitate Immune Escape in LCBM

**Figure 2:**
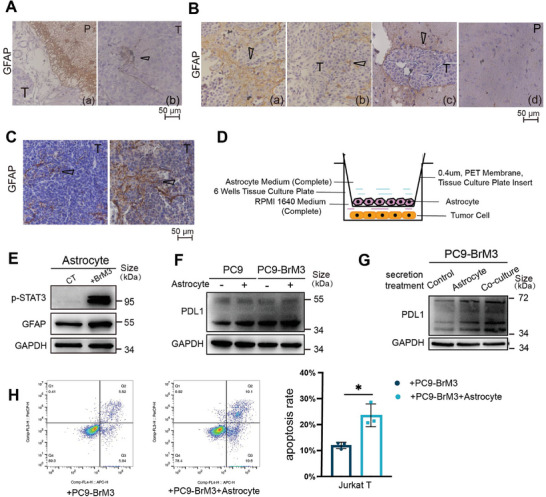
Reactive astrocytes in the brain TME facilitate immune escape in LCBM. A–C) Immunohistochemistry of GFAP (marker of activated astrocytes) in LCBM samples (scale bars: 50 µm). (A) Human sample, showing BM (T) and parenchyma (P), with tumor tissue surrounded by astrocytes (a) and the black arrow showing infiltration of astrocytes into tumor tissue (b), *n* = 5. (B) BALB/c‐nu mouse sample, showing BM (T) and parenchyma (P), with the black arrows showing tumor tissue surrounded by astrocytes (a, b, c) and parenchymal cells distal from the tumor (d), *n* = 10. (C) C57 mouse sample, showing BM (T), with black arrows showing infiltration of astrocytes into tumor tissue (a, b), *n* = 5. D) Design of the co‐culture experimental model of astrocytes and tumor cells constructed using a Tissue Culture Plate insert (0.4 µm, PET membrane: 6 wells). E) Western blotting of p‐STAT3 and GFAP after astrocytes were co‐cultured with or without PC9‐BrM3 cells for 12 h. F) Western blotting of PDL1 in PC9 cells and PC9‐BrM3 cells that were co‐cultured with or without astrocytes for 12 h. G) Western blotting of PDL1 in PC9‐BrM3 cells that were treated with or without secretions for 12 h. H) Flow cytometry results of Jurkat T cells (left) that were co‐cultured with PC9‐BrM3 cells or PC9‐BrM3 cells that were co‐cultured with astrocytes for 24 h, and then harvested for analysis of apoptosis, indicated by dots in the right boxes (Q2+Q3). Quantitation of these results (right), with comparison using an unpaired two‐tailed *t*‐test. Western blotting, and flow cytometry results show representative samples from three or more replicates. Details of the co‐culture process for western blotting and flow cytometry are shown in the Supporting Information and Figure S7 (Supporting Information). *(*p* < 0.05), **(*p* < 0.01), ***(*p* < 0.001), and ****(*p* < 0.0001).

The acquisition of reactive astrocytes, which are characterized by high levels of glial fibrillary acidic protein (GFAP)^[^ ^20^ ^]^ and activation of signal transducer and activator of transcription 3 (STAT3),^[^ ^21^ ^]^ occurs during LCBM. Our results verified an enrichment of reactive astrocytes around BM based on analysis of intratumor infiltration of tissues from clinical samples of BM and an animal model of BM in which PC9‐BrM3 or mouse Lewis lung cancer (LLC) cells were implanted into BALB‐c‐nu or C57 mice, respectively (**Figure** **2**A–C).

We then examined whether the accumulation of astrocytes contributed to the immune escape of BM by establishing a co‐culture system of tumor cells with astrocytes that were transformed into the reactive subtype (Figure 2D,E; Figure S2A, Supporting Information). Analysis of the immunosuppressive phenotypes of tumor cells demonstrated that co‐culture of astrocytes with PC9‐BrM3 cells or PC9 parental cells led to significant upregulation of PDL1, compared with cells that were cultured alone (Figure 2F; Figure S2B, Supporting Information). The results were similar when the *EGFR*‐mutant cell line H1650 (EGFR^Δexon19^ mutation) was co‐cultured with astrocytes, but not in the four cell lines (H1299, A549, H2030, and H2030‐BrM) that had the wild‐type *EGFR* (Figure S2C,D, Supporting Information). Notably, PC9‐BrM3 cells treated with the secretion from the co‐culture system also had greater PDL1 expression than cells treated with the secretion from quiescent astrocytes that were cultured alone (Figure 2G; Figure S2E, Supporting Information). These results suggest that the secretion from reactive astrocytes regulated the tumoral level of PDL1.

Microglia, a major type of glial cell in the BM microenvironment, can exert a significant immune effect, but had little apparent effect on PDL1 expression in BM cells (Figure S2F, Supporting Information). Moreover, our flow cytometry results confirmed that BM cells that were co‐cultured with astrocytes led to an increased apoptosis of T cells (Figure 2H). Taken together, these results demonstrated an enrichment of activated astrocytes in LCBM, and that these astrocytes facilitated immune escape by promoting PDL1 expression in BM cells, which decreased infiltration of BM by T cells.

### 2.3 Astrocytes Promote Immune Escape of LCBM by Increasing IL11 Secretion

**Figure 3:**
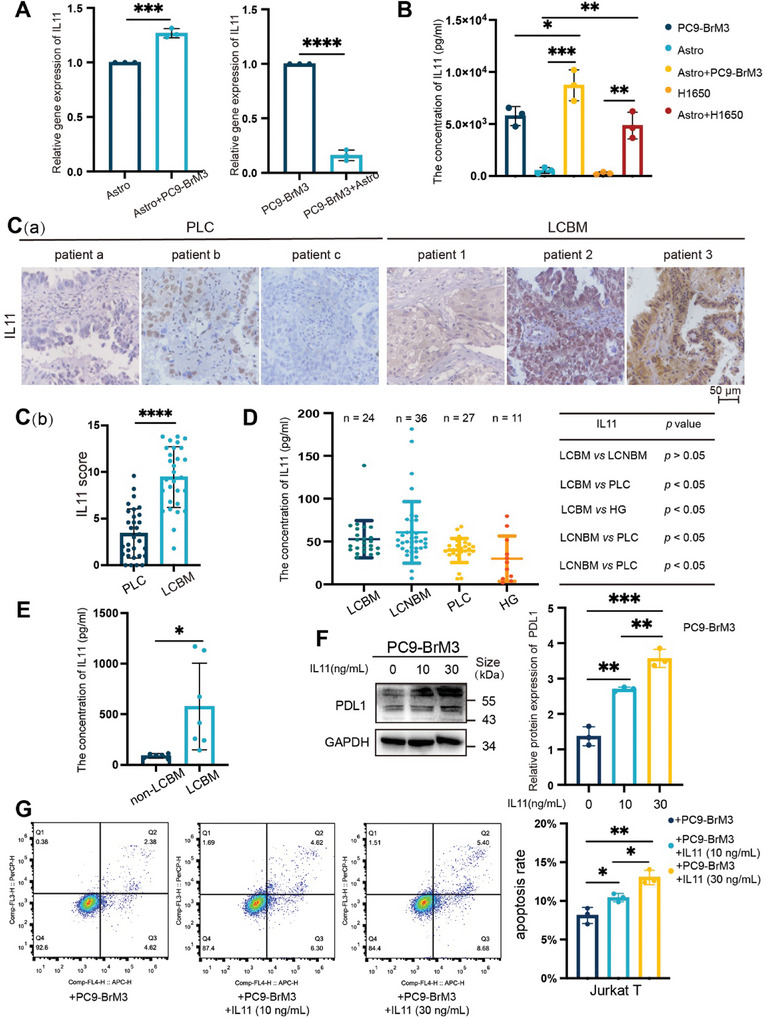
Astrocytes promote immune escape of LCBM by increasing IL11 secretion. A) qPCR of IL11 in astrocytes and PC9‐BrM3 cells that were co‐cultured or cultured separately for 12 h, with comparisons using an unpaired two‐tailed *t*‐test. B) ELISA of IL11 in astrocytes and PC9‐BrM3 or H1650 cells that were co‐cultured or cultured separately for 12 h, with comparisons using an unpaired two‐tailed *t*‐test. C) Immunohistochemistry of IL11 in PLC and LCBM tissues of patients (C[a]), and quantitation of results (C[b], LCBM: *n* = 29; PLC: *n* = 31), with comparison using an unpaired two‐tailed *t*‐test (scale bar: 50 µm). D) ELISA of IL11 levels in serum samples from an LCBM group (*n* = 24), PLC group (*n* = 27), other distant metastases group (LCNBM, *n* = 36) and healthy group (HG, *n* = 11), with comparisons using an unpaired two‐tailed *t*‐test. E) ELISA of IL11 levels in CSF from patients with LCBM (*n* = 7) or without LCBM (*n* = 6), with comparisons using an unpaired two‐tailed *t*‐test. F) Western blotting of PDL1 in PC9‐BrM3 cells that were treated with different concentrations of IL11 (0–30 mg mL^−1^ for 12 h; left), and quantitation of these results (right), with comparison using an unpaired two‐tailed *t*‐test. G) Flow cytometry of Jurkat T cells (left) that were co‐cultured with PC9‐BrM3 cells after treatment with or without IL11 (0–30 ng mL^−1^ for 12 h). Jurkat T cells were harvested for analysis of apoptosis, indicated by dots in the right boxes (Q2+Q3). Quantitation of these results (right), with comparison using an unpaired two‐tailed *t*‐test. Western blotting, qPCR, and flow cytometry results show representative samples from three or more replicates. Details of the co‐culture process for western blotting and flow cytometry are shown in the Supporting Information and Figure S7 (Supporting Information). *(*p* < 0.05), **(*p* < 0.01), ***(*p* < 0.001), and ****(*p* < 0.0001).

We then studied the mechanism responsible for the astrocyte‐induced upregulation of PDL1 in BM cells by using RNA‐sequencing and the tumor cell‐astrocyte co‐culture system (Figure S3A,B, Supporting Information). We identified several Kyoto Encyclopedia of Genes and Genomes (KEGG) pathways that may be associated with the development of LCBM induced by reactive astrocytes. The results showed that the hypoxia response pathway, several pathways related with tumors and tumor metabolism, and some inflammation‐related pathways were up‐regulated in the co‐cultured astrocytes (padj < 0.05, Figure S3C, Supporting Information). Inflammation and immunity are inextricably linked, and tumor‐promoting inflammation is an enabling characteristic of tumors.^[^ ^26^ ^]^ Inflammation can aggravate the accumulation of reactive astrocytes, which promotes tumor progression and the onset of a vicious pathological cycle.^[^ ^27^ ^]^ Therefore, we focused on secretory inflammatory immune molecules among 78 genes with strong significance (padj < 10^−10^) which were up‐regulated in astrocytes co‐cultured with PC9‐BrM3 (Table S3, Supporting Information). The results indicated that IL11, a member of the IL6 family, was significantly up‐regulated in the group with reactive astrocytes, and the intrinsic expression of IL11 in BM cells was decreased after co‐culturing (Figure S3D,E, Supporting Information). The qPCR and ELISA assays confirmed that IL11 was overexpressed and secreted by reactive astrocytes rather than BM cells (**Figure** **3**A,B). Western blotting confirmed that the level of the IL11 protein in co‐cultured astrocytes was also significantly greater (Figure S3F, Supporting Information). IHC examination of IL11 in clinical samples verified a greater level in BM tissues than in PLC tissues (Figure 3C; LCBM: *n* = 29; PLC: *n* = 31). Analysis of the clinical characteristics of these two groups (Table S4, Supporting Information) indicated there were no significant differences in age, gender, or histology (*p* > 0.05). Moreover, the ELISA results indicated that the serum level of IL11 was higher in the LCBM group than in the PLC group and the healthy control group (HG) (Figure 3D, LCBM: n = 24; LCNBM: n = 36; PLC: n = 27; HG: n = 11). The clinical characteristics of the LCBM group are shown in Table S5 (Supporting Information). Notably, these four groups also had no significant differences in age, gender, or histology (Table S5, Supporting Information, *p* > 0.05). Table S6 (Supporting Information) shows the clinical characteristics of the control groups. Importantly, the level of IL11 in the cerebrospinal fluid (CSF) was significantly higher in LCBM patients than in patients with diseases other than NSCLC BM (Figure 3E; Table S7, Supporting Information). These findings indicate that an increased secretion of IL11 by astrocytes within the TME was associated with BM.

We then examined whether IL11 secretion induced immune escape in LCBM by treating PC9‐BrM3 cells with a recombinant IL11 protein. The results showed that IL11 increased the expression of PDL1 in BM cells in a dose‐dependent manner (Figure 3F). The results from the flow cytometry experiment were consistent, in that the apoptosis of Jurkat T cells (induced by PC9‐BrM3) was greater when tumor cells were treated with IL11, especially at a higher concentration (Figure 3G). Collectively, these results indicate that reactive astrocytes had increased secretion of IL11 and that this promoted the immune escape of LCBM.

### 2.4 IL11 Promotes Immune Escape in LCBM by Activation of IL11Rα/gp130 and EGFR

**Figure 4:**
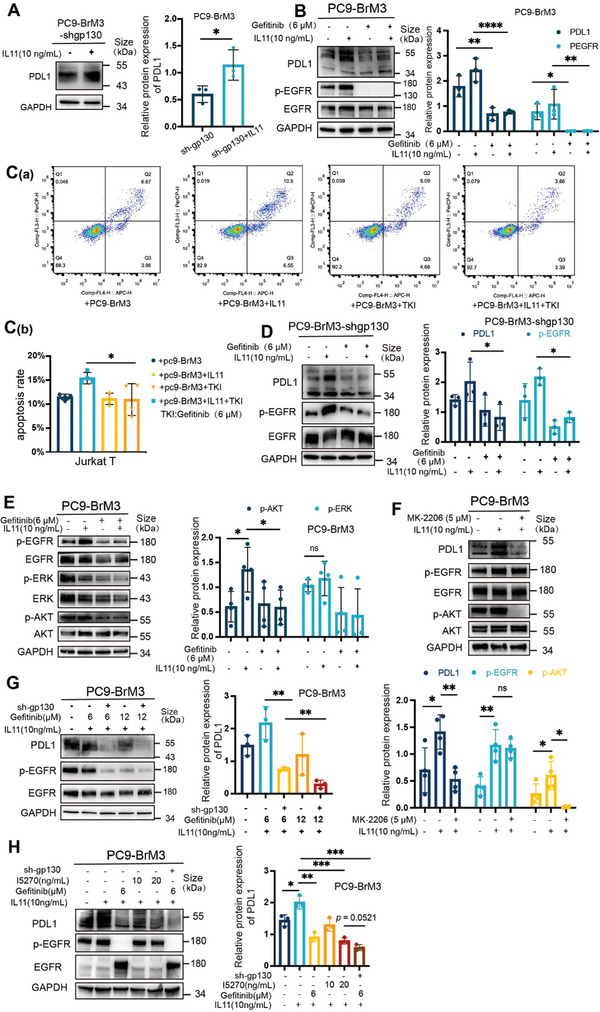
IL11 promotes immune escape in LCBM by activation of IL11Rα/gp130 and EGFR. A) Western blotting of PDL1 in PC9‐BrM3‐shgp130 cells that were treated with or without IL11 (10 ng mL^−1^ for 12 h; left), and quantitation of these results (right), with comparison using an unpaired, two‐tailed *t*‐test. B) Western blotting of PDL1, p‐EGFR, and EGFR in PC9‐BrM3 cells that were treated with or without IL11 (10 ng mL^−1^ for 12 h) and with or without gefitinib (6 µM for 18 h) (left), and quantitation of these results (right), with comparisons using a 2‐way ANOVA with Sidak's multiple comparisons test. C) Flow cytometry of Jurkat T cells (C[a]) that PC9‐BrM3 were treated with or without IL11 (10 ng mL^−1^ for 12 h) and with or without gefitinib (6 µM for 18 h), and then co‐cultured with Jurkat T for 24 h. Jurkat T cells were harvested for analysis of apoptosis, indicated by dots in the right boxes (Q2+Q3). Quantitation of the results (C[b]), with comparisons using an unpaired two‐tailed *t*‐test. D) Western blotting of PDL1, p‐EGFR, and EGFR in PC9‐BrM3‐shgp130 cells that were treated with or without IL11 (10 ng mL^−1^ for 12 h) and with or without gefitinib (6 µM for 18 h; left), and quantitation of these results (right), with comparisons using a 2‐way ANOVA with Tukey's multiple comparisons test. E) Western blotting of p‐EGFR, EGFR, p‐AKT, AKT, p‐ERK, and ERK in PC9‐BrM3 cells that were treated with or without IL11 (10 ng mL^−1^ for 12 h) and with or without gefitinib (6 µM for 18 h; left), and quantitation of these results (right), with comparisons using a 2‐way ANOVA with Tukey's multiple comparisons test. F) Western blotting of p‐EGFR, EGFR, p‐AKT, AKT, and PDL1 in PC9‐BrM3 cells that were treated with or without IL11 (10 ng mL^−1^ for 12 h) and with an AKT inhibitor (MK‐2206, 5 µM for 18 h; top), and quantitation of these results (bottom), with comparisons using a 2‐way ANOVA with Tukey's multiple comparisons test. G) Western blotting of PDL1, p‐EGFR, and EGFR in PC9‐BrM3 cells with or without silencing by shgp130, with or without IL11 (10 ng mL^−1^ for 12 h), and with different concentrations of gefitinib (0–12 µM for 18 h; left), and quantitation of PDL1 expression (right), with comparisons using an unpaired, two‐tailed *t*‐test. H) Western blotting of PDL1, p‐EGFR, and EGFR in PC9‐BrM3 cells with or without silencing by shgp130, with different concentrations of an IL11 neutralizing antibody (I5270; 0–20 ng mL^−1^ for 48 h), with or without gefinitib (6 µM for 18 h), and with or without IL11 (10 ng mL^−1^ for 12 h; left), and quantitation of PDL1 expression (right), with comparisons using an unpaired, two‐tailed *t*‐test. Western blotting and flow cytometry results show representative samples from three or more replicates. Details of the co‐culture process for western blotting and flow cytometry are shown in the Supporting Information and Figure S7 (Supporting Information). *(*p* < 0.05), **(*p* < 0.01), ***(*p* < 0.001), and ****(*p* < 0.0001).

The classical signaling pathway of IL11 begins with binding to IL11Rα and gp130 to form a 2:2:2 hexamer, followed by downstream signaling.^[^ ^28^, ^29^, ^30^ ^]^ When we silenced gp130 in PC9‐BrM3 cells (PC9‐BrM3‐shgp130), it blocked this pathway (Figure S4A, Supporting Information). However, the expression of PDL1 in PC9‐BrM3‐shgp130 cells was still upregulated after treatment with IL11(**Figure** **4**A). This suggested that an additional pathway contributed to the effect of IL11 on LCBM.

The activation of EGFR closely correlated with the immunosuppressive phenotype in LCBM. We found that PC9‐BrM3 cells had a higher level of p‐EGFR than the parent PC9 cells (Figure S4B, Supporting Information); however, the H2030‐BrM cells had a lower level of p‐EGFR compared to the parent H2030 cells (Figure S4C, Supporting Information). Moreover, H1650 cells co‐cultured with astrocytes had higher expression of p‐EGFR and PDL1 than the control group (Figure S4D, Supporting Information). These results suggest that EGFR activation functioned in the IL11‐mediated effects in LCBM. Hence, we pre‐treated PC9‐BrM3 cells with an EGFR‐TKI (gefitinib, 6 µm) to block the EGFR pathway, and then administered IL11. The results showed that gefitinib suppressed the IL11‐mediated upregulation of PDL1 in PC9‐BrM3 cells and in H1650 cells (Figure 4B; Figure S4E, Supporting Information). This treatment also decreased the ability of tumor cells to induce apoptosis in T cells (Figure 4C). In addition, gefitinib suppressed the upregulation of PDL1 in PC9‐BrM3 cells and decreased the percentage of apoptotic Jurkat T cells that were induced by co‐culture of PC9‐BrM3 cells with astrocytes (Figure S4F,G, Supporting Information). Gefitinib also suppressed the upregulation of PDL1 in PC9‐BrM3‐shgp130 cells that were treated with IL11 (Figure 4D). These results suggest that activation of EGFR was necessary for the IL11‐mediated promotion of immune escape in LCBM.

The activation of EGFR also stimulates of extracellular signal‐regulated kinase (ERK) and AKT signaling.^[^ ^31^ ^]^ Our results showed that IL11 administration increased the level of phosphorylated AKT (p‐AKT), the activated form (Figure 4E), and that an AKT inhibitor (MK‐2206) significantly suppressed IL11‐induced upregulation of PDL1, but did not affect the level of p‐EGFR (Figure 4F). The results were similar when using H1650 cells (Figure S4H, Supporting Information). These results indicate that the IL11‐induced increase of PDL1 expression was mediated by the AKT pathway.

Although our results demonstrated that EGFR mediated the IL11‐induced immunosuppressive effect, combining gp130 silencing with gefitinib treatment was more effective than gefitinib alone in suppressing the IL11‐mediated increases of PDL1 (Figure 4G). In addition, there was no difference in p‐EGFR and PDL1 expression following treatment with gefitinib (6 µM) alone and treatment with gefitinib in combination with sh‐gp130 without IL11 (Figure S4I, Supporting Information). This demonstrates that the combined targeting gp130 and EGFR effectively decreased brain metastases that were induced by IL11. Of note, an IL11 neutralizing antibody (I5270) also suppressed the upregulation of PDL1 in PC9‐BrM3 cells that were treated with IL11 (Figure 4H). These findings suggest that IL11Rα/gp130, the classical receptor of IL11, also functioned in the immunosuppressive effect. Collectively, these results demonstrate that IL11 promoted immune escape in LCBM by activation of signaling through the IL11Rα/gp130 and EGFR pathways.

### 2.5 IL11 Binds to EGFR as a Ligand and Stimulates EGFR Phosphorylation

**Figure 5:**
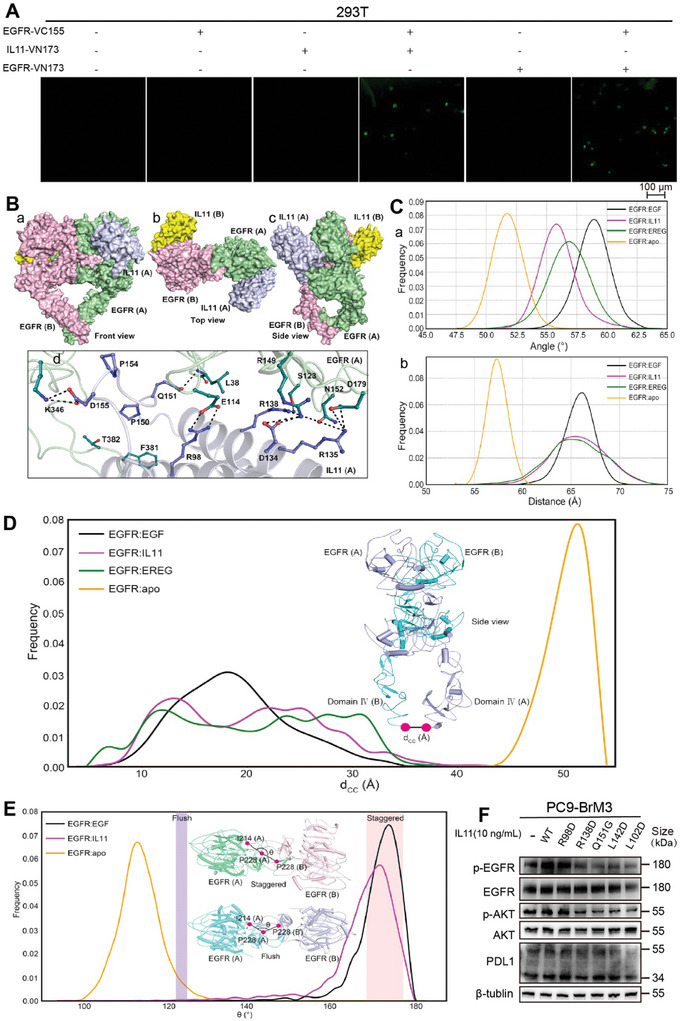
IL11 binds to EGFR as a ligand and stimulates EGFR phosphorylation. A) Fluorescence microscopy of 293T cells that were transfected with different Venus fusion genes (scale bar: 100 µm). B) Front view (a), top view (b), and side view (c) of the interaction of EGFR dimer with two IL11 molecules, showing that the α‐helix barrel of IL11 had a parallel contact pattern with domain I of EGFR, and a cross‐contact pattern with domain III of EGFR. The EGFR monomers are pink and green, and the IL11 molecules are yellow and blue. A magnified view of the interaction between EGFR and IL11 (d), in which sticks show the residues involved in the interactions and stick colors have the same meanings as above. C) Probability distribution of the angles (a) and distances (b) defined in Figure S5D (Supporting Information), respectively. D) Distribution of the distance (d_CC_) formed by the C termini of two the domain IV regions during simulations. E) The major distinction between the staggered mode and flush mode is the angle (θ) formed by the Cα atom of I214 and P228 in one subunit, and the Cα atom of P228 in the other subunit. Calculations showed that removing EGF from the dimer decreased θ from 170° to 115°, indicating a change from the staggered mode to the flush mode. The insert shows top views of the staggered and flush conformations of the EGFR extracellular dimers. F) Western blotting of p‐EGFR, EGFR, p‐AKT, AKT, and PDL1 in PC9‐BrM3 cells that received no treatment (‐), treatment with WT IL11 (10 ng mL^−1^ for 12 h, Supporting Information), or treatment with five different IL11 mutants (10 ng mL^−1^ for 12 h, Supporting Information for 12 h). Western blotting and BiFC results show representative samples from three or more replicates.

Because IL11 induced the phosphorylation of EGFR and this was blocked by gefitinib, we further investigated the interaction between IL11 and EGFR using a bimolecular fluorescence complementation (BiFC) assay. Thus, we constructed a Venus fusion gene, in which the C‐terminus (VC155) was fused to EGFR and the N‐terminus (VN173) was fused to IL11, and then transfected this gene into non‐GFP luciferase labeled 293T cells; the positive control consisted of EGFR‐VC155 and EGFR‐VN173 (Figure S5A,B, Supporting Information). When the VC155 and VN173 domains are in close proximity, this reconstitutes the fluorescence activity of the Venus protein.^[^ ^32^ ^]^ Similar to our positive control group, 293T cells transfected with EGFR‐VC155 and IL11‐VN173 had obvious fluorescence (**Figure** **5**A). These results suggest there was binding of IL11 and EGFR at the cellular level.

We then used molecular dynamics (MD) simulations to further explore the molecular characteristics of the binding of IL11 with EGFR, with a focus on five binding positions (Figure S5C, Supporting Information). The results from the molecular mechanics/generalized Born and surface solvation analysis (MM/GBSA) showed that the free energy of binding (−138.45 kcal mol^−1^) was lowest for EGFR and IL11 at position 4 (Figure S5C, Supporting Information). Therefore, we selected this position for subsequent analysis. Analysis of the spatial structure demonstrated that IL11 was located in the ligand‐binding pocket of EGFR (Figure 5B). We then used the epidermal growth factor (EGF, a ligand with higher affinity) and epiregulin (EREG, a ligand with lower affinity) as positive controls and the apo EGFR dimer (a non‐binding ligand) as a negative control and performed simulations to examine whether IL11 played the same role as the classical ligands in regulating EGFR. The results showed that binding of EREG and IL11 increased the angle of the binding pocket by 56°, slightly less than the increase caused by EGF (59°) (Figure 5C‐a), but slightly more than the increase caused by the apo EGFR dimer (52°). These results suggest that IL11 has low‐affinity for the ligand‐binding pocket of EGFR.

On the other hand, the binding of EREG and IL11 to EGFR increased the distance from Domain II to the C‐terminus of Domain IV from 57.5 Å to 65 Å, less than that caused by EGF (57.5 Å to 66 Å; Figure 5C‐b; Figure S5D, Supporting Information). This indicated that the binding of IL11 also caused the C‐terminus of Domain IV to move away from the other three extracellular domains. Correspondingly, the binding of EGF, EREG, and IL11 decreased the distance between the two C‐terminals (d_CC_) from 50 Å to 20 Å (Figure 5D), meaning that the two C‐terminuses were intertwined and that EGFR was activated.^[^ ^33^ ^]^ Previous research found that removal of EGF from the staggered mode caused a change to the flush mode.^[^ ^33^ ^]^ Therefore, our results showed that the binding of IL11 maintained the dimer in the staggered conformation, suggesting that IL11 binds to the same site of EGFR as EGF, the classical ligand (Figure 5E). Furthermore, analysis of energy decomposition residues (Table S8, Supporting Information) showed that residues F381, L38, E114, R149, T382, D179, N152, K346, and N115 from EGFR and residues R98, R138, Q151, L142, P154, L102, D155, P150, D134, and R135 from IL11 functioned in the interaction between IL11 and EGFR.

We therefore engineered a recombinant IL11, in which several of these sites were mutated, administered the native IL11 or the five different mutated versions to PC9‐BrM3 cells, and measured the cellular level of p‐EGFR. As predicted, the different mutant forms of IL11 lost their ability to activate EGFR to different degrees (Figure 5F). Collectively, these results suggest that IL11 binds to and actives EGFR as a ligand in a manner similar to EGF, its classical ligand, and transforms EGFR into the activated state.

### 2.6 Combined Targeting gp130 and EGFR Inhibits PDL1 Expression and Restores T Cell Infiltration of BM

**Figure 6:**
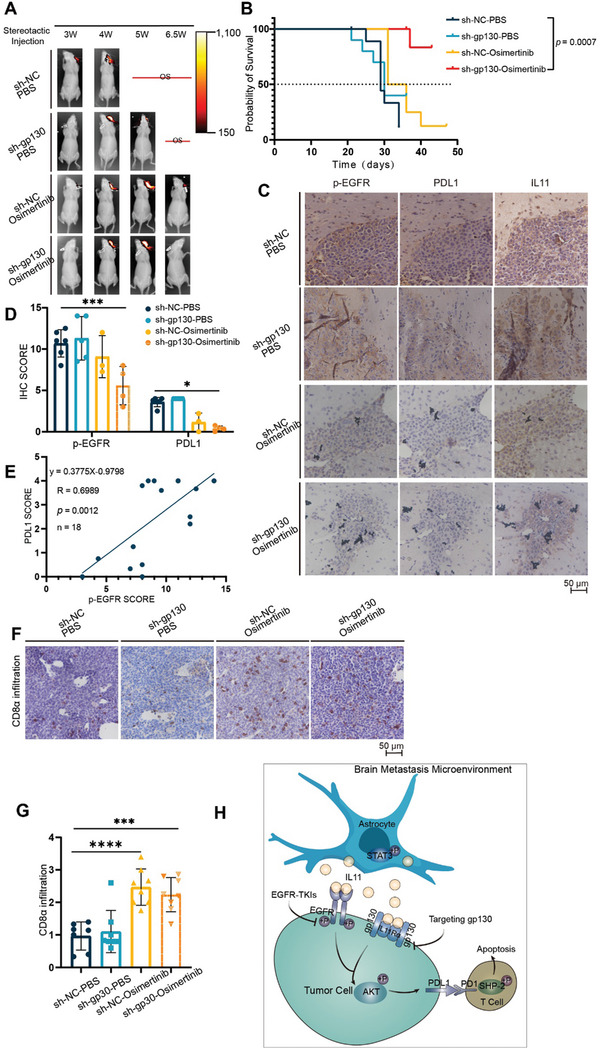
Combined targeting of gp130 and EGFR inhibits PDL1 expression and restores T cell infiltration of brain lesions in mouse models. A) Whole‐body bioluminescence imaging of mice was performed every week beginning the third week after injection of PC9‐BrM3 cells to induce brain lesions. B) Survival curves of mice in the different treatment groups (NC+PBS: *n* = 10; NC+osimertinib: *n* = 8; sh‐gp130+PBS: *n* = 10; sh‐gp130+osimertinib: *n* = 7), with comparison using the log‐rank test. C,D) Immunohistochemistry of PDL1, p‐EGFR, and IL11 in brain lesions of mice that received different treatments (scale bar: 50 µm), and quantitation of staining scores, with comparisons using a 2‐way ANOVA with Tukey's multiple comparisons test. E) Relationship of the expression of PDL1 and p‐EGFR in brain lesions (n = 18, simple linear regression). F,G) Immunohistochemistry of CD8α infiltration of brain lesions in the different treatment groups (scale bar: 50 µm), and quantitation of these results, with comparisons using an unpaired, two‐tailed *t*‐test. H) Proposed mechanism of brain metastasis in *EGFR*‐mutated NSCLC, and blockage by osimertinib (EGFR‐TKI). Reactive astrocytes have high levels of p‐STAT3 and increased secretion of IL11 (top). IL11 binds to EGFR and gp130 on tumor cells, and activation of these receptors leads to phosphorylation of AKT (bottom‐left). P‐AKT increases the expression of PDL1 and induces the apoptosis of T cells (bottom‐right), culminating in immune escape. Osimertinib blocks EGFR activation and immune escape, and combined targeting of gp130 and EGFR inhibits PDL1 expression and immune escape (middle‐left). *(*p* < 0.05), **(*p* < 0.01), ***(*p* < 0.001), and ****(*p* < 0.0001).

Our demonstration that IL11 plays an important role in regulating the immunosuppressive phenotype in the brain TME by activating the gp130 and EGFR pathways suggests that blocking IL11 may alter the brain TME phenotype. Although T‐cell infiltration could not be assessed in immunodeficient mice, we evaluated the therapeutic effect of the combined targeting gp130 and EGFR on PDL1 expression in PC9‐BrM3 cells in vivo. Thus, we administered intracranial injections of PC9‐BrM3 cells with or without gp130 knock‐down to BALB/c‐nu mice, administered osimertinib or PBS, and then performed bioluminescence imaging for seven days beginning on the third week to analyze the formation of intracranial tumors (**Figure** **6**A). Survival analysis indicated that the combined targeting strategy prolonged the survival time in this mouse model of BM (Figure 6B; Figure S6A, Supporting Information). At the end of observation period, we harvested tumor masses and sectioned them for IHC staining. The results showed there was significant infiltration of IL11 in all groups, but the levels of p‐EGFR and PDL1 were significantly lower in the sh‐gp130+osimertinib groups than in the control (PBS) groups (Figure 6C,D). As expected, the PDL1 staining score had a positive correlation with the p‐EGFR score (Figure 6E).

We further evaluated the efficacy of this combined targeting strategy on the restoration of immune cell infiltration in immunized mice. Thus, we generated a mouse‐derived LLC cell line that overexpressed EGFR^Del732‐763aa^ with or without gp130 knock‐down, and then performed intracranial injection to administer these cells to C57 mice. Osimertinib or PBS treatment was given regularly when the body weight loss was 5%, and tumor masses were harvested when the mice had a body weight loss of 20%. The results showed that the infiltration by CD8^+^ T cells was significantly restored in the sh‐gp130+osimertinib group relative to the PBS groups (Figure 6F,G). However, we also found that the groups had no significant differences in survival time or intracranial tumor size (Figure S6B,C, Supporting Information), indicating that other factors affected tumor growth and development in these mouse‐derived tumor cells. Taken together, these results indicate that the combined targeting of signaling via gp130 and EGFR effectively inhibited the expression of PDL1 and restored T cell infiltration into BM.

## 3 Discussion

Patients with *EGFR*‐mutated NSCLC have an increased risk of BM and poor prognosis.^[^ ^5^, ^6^, ^7^ ^]^ Although recent studies showed that osimertinib provided benefits to this patient subgroup, these patients inevitably experience cancer progression due to the development of drug resistance.^[^ ^8^ ^]^ The recent development of ICIs was a milestone in anti‐tumor therapy,^[^ ^34^ ^]^ but ICIs do not provide benefit to patients with *EGFR*‐mutated NSCLC, and therapies that improve the objective response rate (ORR) of these patients are limited.^[^ ^15^ ^]^ Anti‐PD1/PDL1 agents are the most common and widely‐used types of immunotherapies. These therapies target the PDL1 ligand on tumors or the PD1 receptor on T cells, and this leads to activation of effector T cells and prevents tumor immune escape.^[^ ^35^ ^]^ Therefore, it is necessary to identify new biomarkers that provide better predictions of clinical response and can potentially be used as immunotherapy targets for more effective combination therapies that overcome immune resistance.

According to the spatial distribution of cytotoxic immune cells in the TME, a tumor is classified as immune‐inflamed, immune‐excluded, or immune‐deserted.^[^ ^36^ ^]^ The T‐cell‐immune‐inflamed tumor (“hot” phenotype) has been proposed as a predictor of response to ICIs.^[^ ^37^ ^]^ In contrast, immune‐excluded tumors and immune‐deserted tumors have been described as “cold*”* tumors with poor T‐cell infiltration that rarely respond to ICI monotherapy and require combination therapy for increased antitumor efficacy.^[^ ^38^, ^39^ ^]^ However, the diversity of immune escape mechanisms is a major obstacle to the transformation of unresponsive “cold” tumors into responsive “hot” tumors. Therefore, exploring the mechanisms of this transformation and the use of tumor immunophenotyping can provide important insights that may enable the design of precise strategies for targeting tumors. A recent review summarized the mechanisms of the “cold” tumor phenotype and therapies that can be used to improve T‐cell infiltration, and suggested that restoring T cell infiltration may make “cold” tumors more sensitive to ICI therapy.^[^ ^40^ ^]^ The present study explored a novel mechanism of treating “cold” tumors with poor T cell infiltration in TME, and provided a new strategy to restore T cell infiltration. In addition, another study proposed a paradigm of four tumor immunophenotypes based on the tumor immune microenvironment, specifically PDL1 expression and tumor‐infiltrating lymphocytes (TILs): PDL1−/TIL−, PDL1+/TIL+, PDL1−/TIL+, and PDL1+/TIL−.^[^ ^41^ ^]^ This paradigm considered PDL1+/TIL− tumors as resistant to anti‐PD1/PDL1 therapy because of low T cell infiltration. Consistently, our results demonstrated the immune TME of *EGFR*‐mutated NSCLC was characterized by overexpression of PDL1 and decreased infiltration by T cells, consistent with “cold” tumors, and this might explain the poor ORR in patients with *EGFR*‐mutated NSCLC who receive ICIs. We also found that overexpression of PDL1 and decreased infiltration of T cells had linear correlations with the level of p‐EGFR. These results suggest that further study of the mechanism underlying the activation of EGFR and PDL1 expression in BM is crucial for the development of new ICI agents that restore T cell infiltration in patients with *EGFR*‐mutated LCBM.

Various ligands and cofactors in the TME can regulate the activation of EGFR in tumor cells.^[^ ^42^ ^]^ More specifically, the brain TME has an abundance of reactive astrocytes with tissue infiltration.^[^ ^21^ ^]^ Several studies of BM have reported that astrocytes are crucial to the co‐option of blood vessels,^[^ ^21^ ^]^ chemotherapy‐resistance,^[^ ^20^ ^]^ formation of gap junctions,^[^ ^20^ ^]^ and tumor growth.^[^ ^43^ ^]^ Moreover, there is recent evidence that the pSTAT3^+^ subpopulation of reactive astrocytes is critical for BM because of their ability to modify the TME and promote the metastatic process.^[^ ^21^ ^]^ A recent clinical trial found that targeting activated astrocytes by a STAT3 inhibitor (legasil), rather than whole‐brain radiation and standard chemotherapy, led to a 75% increase of the ORR and a prolonged survival time in patients who had lung cancer with BM.^[^ ^21^ ^]^ Our results showed that pSTAT3^+^‐activated astrocytes promoted immune escape in LCBM by up‐regulating IL11. This provides a new rationale for use of anti‐STAT3 regimens as an immunoadjuvant therapy for treatment of LCBM.

IL11 is a member of IL6 family whose classic receptor is gp130, a transmembrane glycoprotein.^[^ ^44^ ^]^ At the physiological level, IL11 induces platelet maturation in macrophages and increases the platelet count, so IL11 treatment is a common clinical treatment for thrombocytopenia associated with chemotherapy and for promotion of platelet production. Recent studies also found that IL11 plays an important role in the onset and progression of various types of cancer, especially colorectal cancers in which fibroblasts have high expression of IL11.^[^ ^45^, ^46^ ^]^ More importantly, IL11 promotes tumor growth by inhibiting the effect of CD4+ T helper 1 (Th1) cells in tumors, suggesting that IL11 affects tumor immunity.^[^ ^47^ ^]^ Our results showed that IL11 increased PDL1 expression and enhanced the ability of LCBM cells to induce T cell apoptosis after binding to its classical receptor (IL11Rα/gp130), and also by binding to and activating EGFR (Figure 6H). Our molecular modeling and other results also demonstrated that IL11 functioned as a ligand of EGFR, and that this binding maintained EGFR in the active conformation.

Our in vitro and in vivo experiments showed that the combined targeting of gp130 and EGFR by inhibition of IL11 led to decreased PDL1 expression in tumor cells and restoration of the infiltration of T cells, suggesting a potential immunotherapeutic efficacy of this dual blockade strategy for patients with LCBM. A limitation of our approach is that we targeted gp130 by endogenous gene editing rather than with an exogenous inhibitor. However, we used I5270, an antibody that neutralizes human IL11, to verify the inhibition of EGFR phosphorylation and PDL1 expression in tumor cells in vitro. The use of I5270 in animal models is problematic because this antibody targets human IL11, not mouse IL11. A recently described IL11 antagonist, IL11‐mutein, has a binding affinity to IL11Rα that is 20‐times higher than IL11. This new agent can reduce tumor size without affecting platelet and coagulation function in mouse colorectal cancer models.^[^ ^48^, ^49^ ^]^ We therefore suggest that future studies should consider the in vivo application of this IL11 antagonist. Although EGFR‐TKIs efficiently inhibit EGFR activation, drug resistance remains an inevitable problem. Moreover, a recent study showed that the acquisition of this resistance further increased PDL1 expression in tumor cells.^[^ ^50^ ^]^ This indicates a need to identify additional therapies that can effectively inhibit PDL1 and suppress immune escape. Because we demonstrated that IL11 upregulated PDL1 expression by activating the EGFR and gp130 pathways, a combination therapy may be more effective in overcoming resistance to TKIs.

## 4 Experimental Section

Methods describing the specific procedures used for western blotting, Flow cytometrys, quantitative PCR (qPCR), ELISA assays are in the SUPPORTING INFORMATION.

### Materials

*Antibodies*: Primary antibodies were used against: PDL1 (ab205921, Abcam,1:1000 for WB, 2 µg mL^−1^ for IHC); phospho‐EGF Receptor (3777, Cell Signaling Technology,1:1000 for WB, 1:400 for IHC); CD8α (AB4055, Abcam, 1:200 for IHC and IF); CD8α (ab217344, Abcam, 1:2000 for IHC); CD3 (17617‐1‐AP, Proteintech, 1:500 for IHC); GFAP (16825‐1‐AP, Proteintech, 1:2000 for WB, 1:500 for IHC); GAPDH (140494‐1‐AP, Proteintech, 1:5000 for WB); phospho‐STAT3 (310 019, Zenbio, 1:500 for WB); IL11 (55169‐1‐AP, Proteintech, 1:150 for IHC); IL11 (A1902, ABclonal, 1:1000 for WB); gp130(CD130) (ab283689, Abcam, 1:1000 for WB); EGFR (18986‐1‐AP, Proteintech, 1:500 for WB); phospho‐ERK1/2 (8871, Cell Signaling Technology, 1:1000 for WB); ERK1/2 (5903, Cell Signaling Technology, 1:1000 for WB); AKT(pan) (4521, Cell Signaling Technology, 1:1000 for WB) and phospho‐AKT (1482, Cell Signaling Technology, 1:1000 for WB). Two secondary antibodies were used: HRP‐conjugated Affinipure goat‐anti‐rabbit IgG (SA00001‐2, Proteintech, 1:5000 for WB) and CoraLite488‐conjugated Goat Anti‐Rabbit IgG(H+L) (SA00013‐2, Proteintech, 1:100 for IF).

*Reagents*: The CoraLite594 TUNEL Assay Apoptosis Detection Kit was bought from Proteintech. The Pierce BCA Protein Assay Kits were obtained from Thermo Scientific. IL11 recombination protein, Osimertinib, MK‐2206, PEG300, and Tween 80 were purchased from MedChemExpress (*MCE*). The Annexin V‐APC/7‐AAD Apoptosis Detection Kit was from Elabscience. Human IL11 neutralizing antibody (I5270) was from SIGMA. Gefitinib was from Meilunbio.

### Immunohistochemistry and Immunofluorescence

Sections from paraffin‐embedded human or mouse tissues were stained with antibodies as indicated in the text. PDL1 expression was evaluated using the tumor proportion score (TPS),^[^ ^51^ ^]^ which was scored as 0 (<1% TPS), 1 (1–4.9% TPS), 2 (5–9.9% TPS), 3 (10–49.9% TPS), or 4 (≥50% TPS). For CD3 and CD8 staining scores, the level of T cell infiltration of stroma in sections was analyzed using a scale from 0 (low) to 4 (high). Multiple representative fields per tissue section were randomly selected and evaluated under microscopy (×200), and the average score of positive staining in these fields was presented. Other tissue sections were quantitatively scored as the product of the scores for staining intensity and staining percentage (range: 0–15). For this calculation, the score for staining intensity was 0 (negative), 1 (weak), 2 (moderate), or 3 (strong); the score for staining percentage was 0 (<1%), 1 (1–25%), 2 (25–50%), 3 (50–75%), or 4 (75–100%). For evaluation of H&E staining results, all images were acquired by scanning slides with an automated, high capacity, digital pathology slide scanner (Leica, Aperio GT 450).

### Cells and Cell Culture

Human lung adenocarcinoma cell lines (PC9, H2030, H1299, A549), human embryonic kidney cell line 293T, and human microglial cell line HMC3 were from the Chinese Academy of Medical Sciences (Beijing, China). Brain metastatic derivatives (PC9‐BrM3 and H2030‐BrM) were previously constructed by our research group.^[^ ^25^ ^]^ Human lung adenocarcinoma cell lines H1650 and mouse Lewis lung cancer (LCC) cells were from Pricella (Wuhan, China). The human T lymphoblastic leukemia cell line Jurkat T was from Beyotime (Shanghai, China). The human PC9, H2030, H1299, A549, Jurkat T, and brain metastatic derivatives (PC9‐BrM3 and H2030‐BrM cells) were cultured in RPMI 1640 medium (Meilunbio) with 10% fetal bovine serum (FBS) and 1% P/S. Human 293T cells and mouse LLC cells were cultured in DMEM (Meilunbio) with 10% FBS, 1% P/S, and 2 mM L‐glutamine. Human microglial HMC3 cells were cultured in MEM with 10% FBS and 1% P/S. Human HA1800 astrocytes were purchased from ScienCell (USA) and cultured in the medium recommended by the manufacturer; cells from passages 2 to 6 were used for experiments.

All cells tested negative for *Mycoplasma* contamination and were tested by short tandem repeat (STR) analysis at the time of purchase. All cells were pretreated with *Mycoplasma* prophylaxis before cryopreservation, and were then cultured without prophylaxis for 5 to 7 days after cell resuscitation to ensure that the prophylaxis had no effect on the results. All cell lines were maintained in a humidified atmosphere with 5% CO_2_ at 37°C.

### TUNEL Staining

For TUNEL staining, sections from paraffin‐embedded human tissues were stained using CoraLite594 TUNEL Assay Apoptosis Detection Kit (Proteintech, China), according to the manufacturer's protocol. Tissues were visualized using a Leica TCS SP5II confocal laser scanning microscope.

### Cancer Cells and Astrocyte Co‐Culture

Astrocytes and tumor cells were planted in the upper or lower layers of the transwell, respectively. Co‐culture experiments were performed when cell fusion was 90% for astrocytes and 60% for cancer cells. The duration of co‐culture was 12 h, and used a polyethylene terephthalate (PET) membrane (pore size: 0.4 µm) Tissue Culture Plate Insert (Figure 2D). For RNA‐sequencing, cells were digested by Trizol, sequencing was performed by Novogene Co., LTD, and analysis of the results was performed using the NovoMagic after‐sales platform. For ELISA assays, secretions from the upper and lower layers were collected, centrifuged, and supernatants were then analyzed.

### Animal Studies

*BALB‐c‐nu Mice*: Athymic female BALB‐c‐nu mice that were 4 to 6‐weeks‐old were purchased from Beijing Vital River Laboratory Animal Technology Co., Ltd (China). Prior to stereotaxic injection into the brain, 3 to 5×10^5^ luciferase‐labeled PC9‐BrM3 or PC9‐BrM3‐shgp130 cells were suspended in 3 µL of PBS and maintained on ice. After anesthetizing a mouse with a tribromoethanol solution (10 mL kg^−1^ body weight; Sigma, USA), the mouse was fixed in a prone position, the scalp was cut, and then the skull was punctured 0.8 mm to the right and 0.8 mm behind the bregma. Then, a 3 µL cell suspension was slowly injected into to a depth of 2 mm using a microinjector (Hamilton, Switzerland). After 3 to 5 min, the microinjector was slowly removed, the scalp was sutured, and mouse was resuscitated on a heated pad.

Brain colonization was analyzed in vivo using bioluminescence imaging (BLI). After intraperitoneal injection of D‐Luciferin (150 mg kg^−1^ body weight; Promega, USA), images were acquired with an IVIS Spectrum Xenogen system (PerkinElmer, USA) every 7 days beginning 3 weeks after the injection. Living Image software version 2.50 was used to analyze the bioluminescence images. The effects of regular gavage with PBS or osimertinib (5 mg kg^−1^ body weight/day) for 14 days were determined by analysis of four treatment groups (NC+PBS, *n* = 10; NC+OSI, *n* = 8; sh‐gp130+PBS, *n* = 10; and sh‐gp130+OSI, *n* = 7), where NC indicates normal control, sh‐gp130 is the construct, and OSI is osimertinib. Prior to administration, osimertinib was diluted using PEG300, Tween 80, and saline according to the supplier's instructions. PBS was diluted in the same amount of solvent for the control groups. The endpoints were a weight loss of 20% or death. All mice that reached the end point were anesthetized for euthanasia and perfused with PBS. The brain tissues were obtained and fixed with 4% paraformaldehyde for more than 36 h, embedded in paraffin, and sectioned prior to analysis.

*C57BL/6N Mice*: Female C57BL/6N mice that were 4 to 6‐weeks‐old were purchased from Beijing Vital River Laboratory Animal Technology Co., Ltd. (China). Prior to stereotaxic injection into the brain, 3 to 5×10^5^ LLC cells that overexpressed mutant EGFR (EGFR^Del732‐763aa^), with or without knock‐down of gp130, were suspended in 3 µL of PBS and maintained on ice. The stereotaxic injection procedure was same as described above. Subsequent osimertinib or PBS treatment was given regularly when there was 5% weight loss. Similar to above, the four treatment groups were: NC+PBS (n = 10), NC+OSI (*n* = 11), sh‐gp130+PBS (*n* = 9), and sh‐gp130+OSI *(n* = 10). The methods for acquisition and processing of brain tissues were also the same as described above.

### Bimolecular Fluorescence Complementation

The yellow fluorescent protein Venus was employed for BiFC experiments.^[^ ^32^ ^]^ The fusion of the C‐terminus (VC155, Addgene: pBiFC‐VC155) with EGFR and the N‐terminus (VN173, Addgene: pBiFC‐VN173) with IL11 was performed by Shanghai Genechem Co., Ltd. This construct was transfected into non‐GFP luciferase labeled 293T cells in a 96‐well plate, and the specific cell line combinations used are indicated in the figure legends. Fluorescent images were visualized using a Nikon C‐SHG1 fluorescence microscope.

### Molecular Dynamics Simulations

*Preparation of the Protein Structures*: The extracellular crystalline structure of EGFR binding with EGF and EREG was obtained from Protein Data Bank (https://www.rcsb.org) with the PDB ID: 3NPJ for EGFR/EGF complex and 5WB7 for EGFR/EREG complex. While the initial structure of human IL11 was obtained from the AlphaFold Protein Structure Database (https://alphafold.ebi.ac.uk, AF‐P20809‐F1), and it was followed by deleting the residues M1‐P33. The conformation of apo dimer EGFR was constructed by removing the EGF for the EGFR/EGF complex. Subsequently, the IL11 was docked to the apo dimer EGFR by ZDOCK program to build the five possible EGFR/IL11 complexes with highest scores. Because no research has reported the interaction position of IL11 and EGFR, no specific binding position of IL11 was set on EGFR.

*Molecular Dynamics Simulations*: Atomistic MD simulations of EGFR binding with different ligands (EGF, EREG, and IL11) and apo dimer EGFR were carried out using AMBER16 software, embracing Amber ff14SB force field for protein. Each simulation complex was immersed in a hexahedral box of TIP3P water solvent, which was accompanied by a distance of 10 Å from the edge of the water box to the surface of the protein. Counter ions (sodium chloride) were added to the systems to make them behave in a neutral manner. Energy minimization was conducted by imposing a strong restraint on simulations system and was followed by minimizing whole system for a few thousands steps. The NPT ensemble with a temperature of 300 K and a pressure of 1 atm was employed. Prior to the simulation, the models were made more flexible. Subsequently, the production run was performed for 1us with a time step of 4 fs, and the coordinates for all models were saved every 2 ps (each simulation system was repeated for five times). During the production run, the SHAKE algorithm was employed to constrain all bonds associated with hydrogen atoms. Electrostatic interaction was treated by Particle Mesh Ewald and the cutoff value of nonbonded interactions is set to 9 Å.

*The Calculation of Binding Free Energy*: The binding free energy between EGFR with different ligands (EGF, EREG, and IL11) and the energy contribution of each residue to the binding free energy were calculated with MM‐GBSA method base on the 500 snapshots extracted from the last 200 ns MD trajectory. The MM‐GBSA performed in AMBER16, and the electrostatic free energy of solvation (∆G_GB_) was calculated by solving the GB equations computing the binding free energies of the complex systems. The binding energy (∆G_GBTOT_) can be represented as follows:

(1)

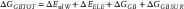

where ∆G_GBTOT_ was obtained by summing the nonpolar (∆G_GBSUR_), the van der Waals (∆E_vdW_) energies, the contributions polar (∆G_GB_) and electrostatic energy (∆E_ELE_). The ∆G_GB_ was calculated by solving the GB equation, while ∆E_ELE_ and ∆E_vdW_ were calculated according to the AMBER ff14SB.

### Knockdown,Overexpression, and IL11 Mutation Protein Constructs

The shRNAs in lentiviral vector for stable knockdown of gp130 and the lentiviral vector for stable expression of EGFR^Del732‐763aa^ mutation were constructed by Shanghai Genechem Co., Ltd. The infection assays were performed according to the manufacturer's protocol. IL11 mutation protein WT, R98D, R138D, Q151G, L142D, and L102D were from HUABIO (China). The gene sequences and amino acid sequences are listed in the Table S9 (Supporting Information).

### Statistics

All statistical analyses were performed using GraphPad Prism version 10, and the specific statistical tests are indicated in the figure legends. Western blotting, immunohistochemistry, and flow cytometry results show representative samples from three or more replicates, and bar charts show means ± standard deviations (SDs) from three or more replicates. IBM Statistical Package for the Social Sciences (SPSS) version 25.0 (IBM Corp, Armonk, NY, USA) was used to identify statistical differences between groups. The data of different groups were compared using analysis of variance (ANOVA), the *t*‐test, or the chi‐square test. A *p‐*value below 0.05 was considered significant, and different *p*‐values are indicated by *(*p* < 0.05), **(*p* < 0.01), ***(*p* < 0.001), and ****(*p* < 0.0001).

### Ethics Approval Statement

All animal experiments involving the use of animals were approved by Dalian Medical University Licensing Committee. Ethical project number:*No*. 001 22773 & AEE23117.

### Patient Consent Statement

Written informed consent was obtained from all participants. The use of human NSCLC and LCBM tissues, serums, and CSF from the Second Hospital of Dalian Medical University was approved by the Ethics Review Committee of the Second Hospital of Dalian Medical University. Project number: DYEY‐2020‐020.

## Conflict of Interest

The authors declare no conflict of interest.

## Supporting information

Supporting Information

## Data Availability

The RNA sequencing raw data has been deposited in NCBI GEO with accession number GSE242073. Furthermore, the data that support the findings of BiFC and MD simulations were listed in the corresponding Methods.
