# Supplementary material for: Brain Metastasis from EGFR‐Mutated Non‐Small Cell Lung Cancer: Secretion of IL11 from Astrocytes Up‐Regulates PDL1 and Promotes Immune Escape
Source: Adv Sci (Weinh). 2024 May 2;11(26):2306348. doi: 10.1002/advs.202306348 (PMC11234401; doi:10.1002/advs.202306348)
Supplement: Supplementary file 1 — Supporting Information [file ADVS-11-2306348-s001.pdf]

ADVANCED SCIENCE
Open Access

Supporting Information

for *Adv. Sci.*, DOI 10.1002/advs.202306348

Brain Metastasis from *EGFR*-Mutated Non-Small Cell Lung Cancer: Secretion of IL11 from Astrocytes Up-Regulates PDL1 and Promotes Immune Escape

Mengyi Tang, Mingxin Xu, Jian Wang, Ye Liu, Kun Liang, Yinuo Jin, Wenzhe Duan, Shengkai Xia, Guohui Li, Huiying Chu, Wenwen Liu* and Qi Wang**

Supplementary methods

Drugs and cells preconditioning for western immunoblotting

Tumor cells (PC9, PC9-BrM3) were seeded in six-well plates for 24h and then treated with or without astrocytes (12 h)/IL11 (10 or 30 ng/mL, 12 h)/gefitinib (6 or 12 μM ,18 h)/MK-2206 (5 μM ,18 h)/I5270(10 or 20 ng/mL, 48 h). H1650 cells were seeded in six-well plates for 24h and then treated with or without astrocytes (12 h)/IL11 (20 ng/ml, 12 h)/gefitinib (6 μM ,18 h)/MK-2206 (5 μM ,18 h). Two schematics of drugs and cells preconditioning were shown as Figure S7A-B.

Western immunoblotting analysis

Extraction of proteins from cultured cells with RIPA Lysis Buffer (meilunbio®, China) was followed by protein purification with Pierce™ BCA Protein Assay Kits (Thermo Scientific™). The immunoblotting with corresponding antibodies was described in the figure legends.

Flow cytometry

PC9 and PC9-BrM3 cells were treated with or without astrocytes(12h)/IL11 (10 or 30 ng/ml, 12h)/gefitinib (6 μmol/ml ,18h). Then cells were harvested and seeded in six-well plates for 12h. After discarding the medium, Jurkat T cells with 3ml medium were mixed in the wells at a ratio of 1:4 (Jurkat T cells: tumor cells) for 24h. Then Jurkat T cells were stained by the Annexin V-APC/7-AAD Apoptosis Detection Kit (Elabscience®, China) according to the manufacturer's protocol. The stained cells were analysed using flow cytometry (BD Accuri™ C6 Plus Flow Cytometer). The results were analyzed using Flowjo VX. The schematic of drug and cells

preconditioning were shown as Figure S7C.

Quantitative PCR (qPCR)

Total RNA was extracted with SevenFast Total RNA Extraction Kit for Cells (Seven, China). The cDNA was obtained by *TransScript*® One-step gDNA Removal and cDNA SynthesisSuperMix (TransGen Biotech, China). Quantitative PCR analysis was performed using a PerfectStart Green qPCR SuperMix (TransGen Biotech, China). Data were normalized to expression of a control gene (GAPDH) for each experiment. The specific primer sequences were as follows: IL11-forward, 5 '-GAACTGTGTTTGCCGCCTGG-3'; reverse, 5 '-GTCTGGGGAAACTCGAGGGG-3'.

ELISA assays

Human IL11 ELISA KITs were bought from WUHAN HUAMEI BIOTECH Co., Ltd (China) and ELK Biotechnology CO.,LTD (China). The assays were carried according to the manufacturer’s protocol. Written informed consent was obtained from all participants. The use of serum and cerebrospinal fluid from the Second Hospital of Dalian Medical University was approved by the Ethics Review Committee of the Second Hospital of Dalian Medical University.

Supplementary figures

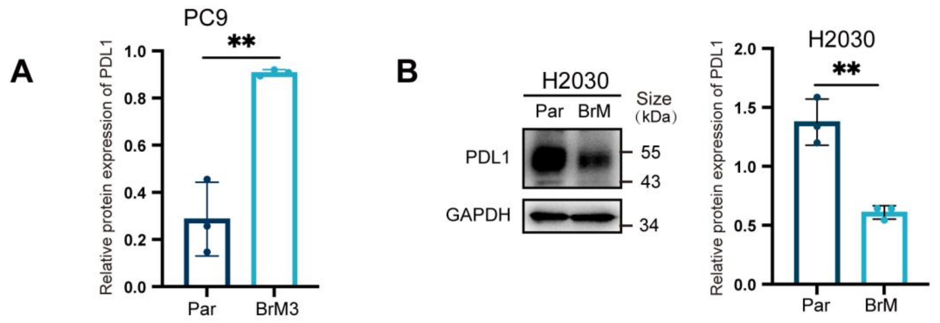

Figure S1 BM from *EGFR*-mutated NSCLC has a stronger immunosuppressive phenotype.

(A) The bar graph was the result of statistical analysis for quantitative scoring of PDL1 protein expression in PC9 and PC9-BrM3 (Unpaired, two-tailed *t*-test). **(B)** Western blotting of PDL1 in H2030(*EGFR* WT) or H2030-BrM (*EGFR* WT) cells (left), and quantitation of these results (right), with comparison using an unpaired, two-tailed *t*-test. Western blotting results show representative samples from three or more replicates. * ($p < 0.05$), ** ($p < 0.01$), *** ($p < 0.001$), and **** ($p < 0.0001$).

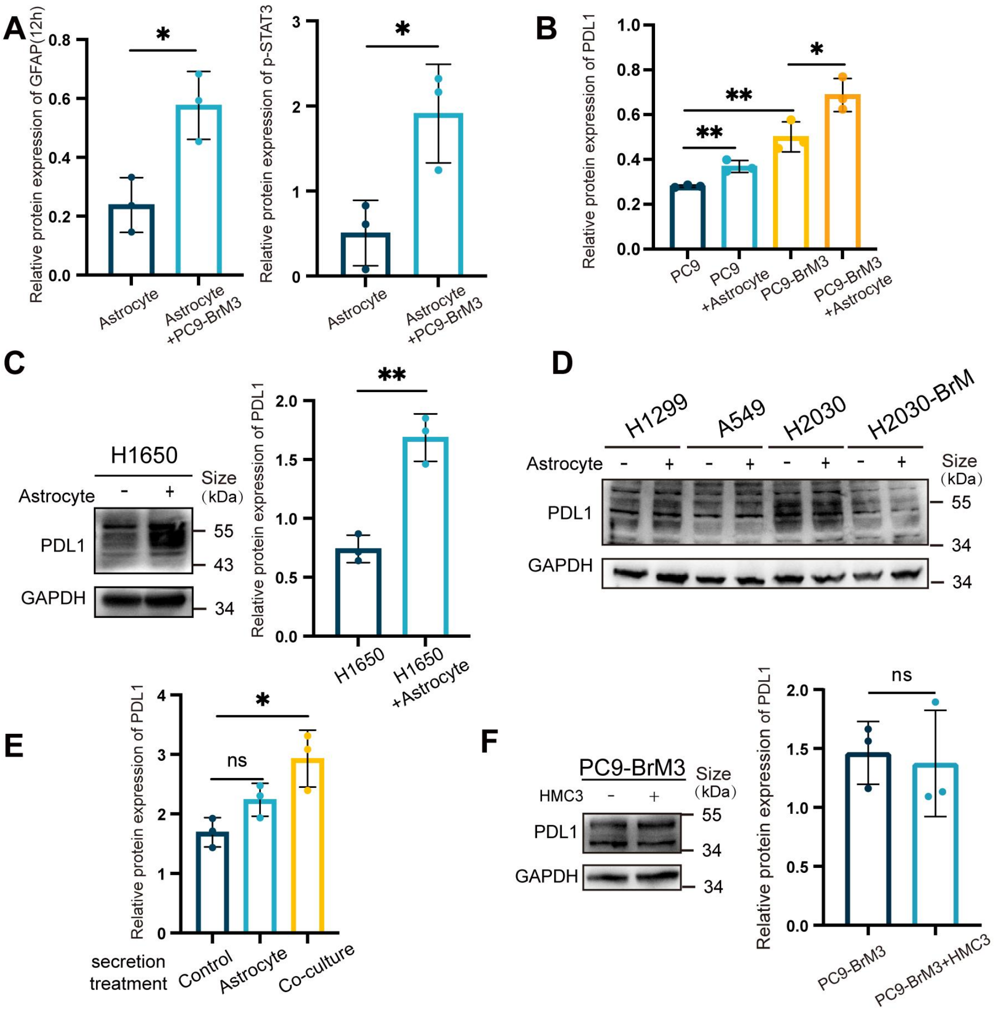

Figure S2 Reactive astrocytes in the brain TME facilitate immune escape in LCBM.

(A) The bar graph was the result of statistical analysis for quantitative scoring of GFAP and p-STAT3 protein expression in astrocytes (Unpaired, two-tailed *t*-test). **(B)** The bar graph was the result of statistical analysis for quantitative scoring of PDL1 protein expression in PC9 and PC9-BrM3 cells (Unpaired, two-tailed *t*-test). **(C)** H1650 cells were co-cultured with or without astrocytes for 12 h. Western blotting of PDL1 in H1650 cells (left), and quantitation of these results (right), with comparison using an unpaired, two-tailed *t*-test. **(D)** EGFR WT cell lines (H1299, A549, 2030 and H2030-BrM) were co-cultured with or without astrocytes for 12 h. Cells were harvested to perform western immunoblotting for PDL1 expression. **(E)** The bar graph was the result of statistical analysis for quantitative scoring of PDL1 protein expression (Unpaired, two-tailed *t*-test). **(F)** PC9-BrM3 cells were co-cultured with microglia for 48h. Western blotting of PDL1 in PC9-BrM3 cells (left), and quantitation of these results (right), with comparison using an unpaired, two-tailed *t*-test. Western blotting results show

representative samples from three or more replicates. * ($p < 0.05$), ** ($p < 0.01$), *** ($p < 0.001$), and **** ($p < 0.0001$).

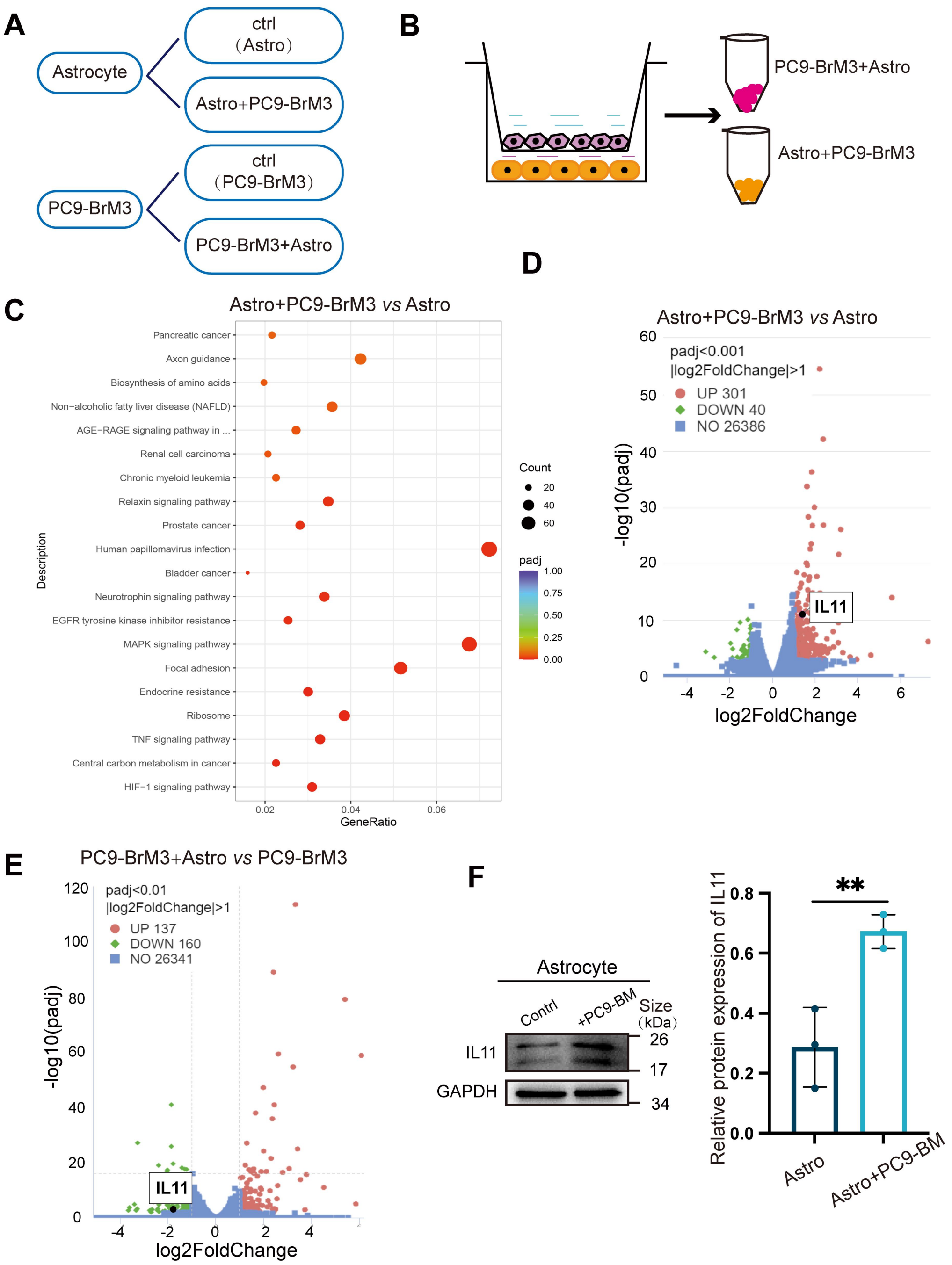

Figure S3 Astrocytes promote immune escape of LCBM by increasing IL11 secretion. **(A-B)** Design of the co-culture experimental groups for RNA-sequences. Astrocytes were co-cultured with or without PC9-BrM3 cells for 12 h. Astrocytes and PC9-BrM3 cells were harvested to conduct RNA-sequences. **(C)** KEGG scatter diagram of differential genes. The abscissa is the ratio of the number of differential genes annotated to the KEGG pathway to the total number of differential genes, and the ordinate is the KEGG pathways. **(D-E)** Differential gene sets of astrocytes or PC9-BrM3 cells before or after co-culture, respectively. The

abscissa is log2FoldChange, and the ordinate is -log10(pvalue). The results of differential gene screening were passed through Novomagic after-sales platform. (C) Astrocytes; (D) PC9-BrM3 cells. **(F)** Astrocytes were co-cultured with or without PC9-BrM3 cells for 12 h. Western blotting of IL11 in astrocytes (left), and quantitation of these results (right), with comparison using an unpaired, two-tailed *t*-test. Western blotting results show representative samples from three or more replicates. * ($p < 0.05$), ** ($p < 0.01$), *** ($p < 0.001$), and **** ($p < 0.0001$).

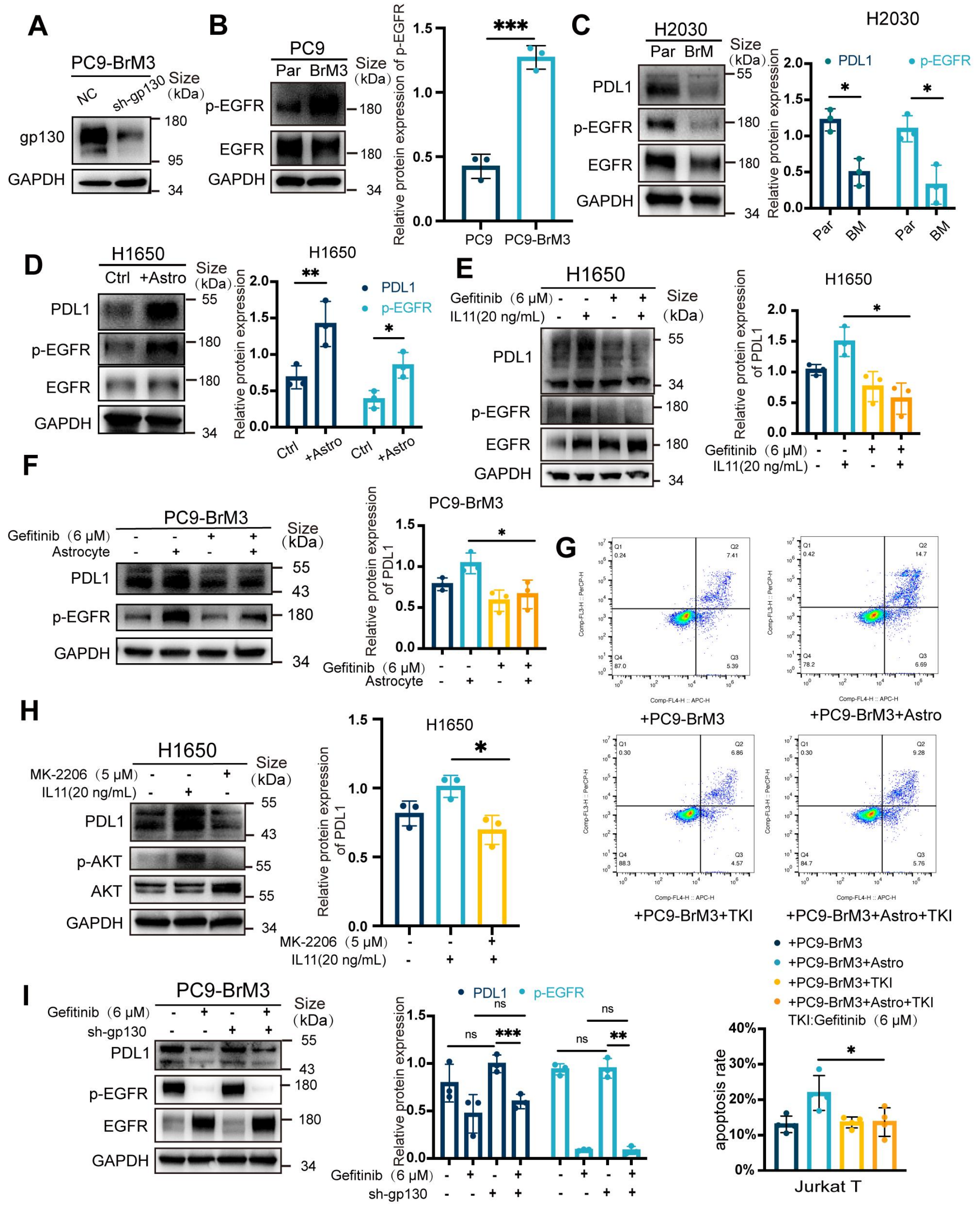

FIGURE S4 IL11 promotes immune escape in LCBM by activation of IL11Rα/gp130 and EGFR.

(A) Gp130 was silenced in PC9-BrM3 cells and cells were harvested to perform western immunoblotting for gp130 expression. **(B)** Western blotting of p-EGFR, and EGFR in PC9 and PC9-BrM3 cells (left), and quantitation of p-EGFR expression (right), with comparison using an unpaired, two-tailed *t*-test. **(C)** Western blotting of PDL1, p-EGFR, and EGFR in H2030 and H2030-BrM cells (left), and quantitation of these results (right), with comparison using a 2-way ANOVA with Sidak's multiple comparisons test. **(D)**H1650 cells were

co-cultured with or without astrocytes for 12 h. Western blotting of PDL1, p-EGFR, and EGFR in H1650 cells (left), and quantitation of these results (right), with comparison using a 2-way ANOVA with Sidak's multiple comparisons test. **(E)** Western blotting of PDL1, p-EGFR, and EGFR in H1650 cells that were treated with or without IL11 (20 ng/mL for 12 h) and with or without gefitinib (6 μM for 18 h; left), and quantitation of PDL1 expression (right), with comparisons using an unpaired two-tailed *t*-test. **(F)** Western blotting of PDL1, and p-EGFR in PC9-BrM3 cells that were treated with or without astrocytes for 12 h and with or without gefitinib (6 μM, 18h; left), and quantitation of PDL1 expression (right), with comparison using an unpaired, two-tailed *t*-test. **(G)** Flow cytometry of Jurkat T cells (top) that PC9-BrM3 were co-cultured with or without astrocytes (12 h) and with or without gefitinib (6 μM for 18 h), and then co-cultured with Jurkat T for 24 h. Jurkat T cells were harvested for analysis of apoptosis, indicated by dots in the right boxes (Q2+Q3). Quantitation of the results (bottom), with comparisons using an unpaired two-tailed *t*-test. **(H)** Western blotting of p-AKT, AKT, and PDL1 in H1650 cells that were treated with or without IL11 (20 ng/mL for 12 h) and with or without an AKT inhibitor (MK-2206, 5 μM for 18 h; left), and quantitation of PDL1 expression (right), with comparisons using an unpaired two-tailed *t*-test. **(I)** Western blotting of PDL1, p-EGFR, and EGFR in PC9-BrM3 cells with or without silencing by shgp130, with or without gefitinib (6μM, 18 h; left), and quantitation of the expression of PDL1 (right), with comparisons using Tukey's multiple comparisons test. Western blotting, and flow cytometry results show representative samples from three or more replicates. Details of the co-culture process for western blotting and flow cytometry are shown in the **Supporting Information** and **Figure S7**. * ($p < 0.05$), ** ($p < 0.01$), *** ($p < 0.001$), and **** ($p < 0.0001$).

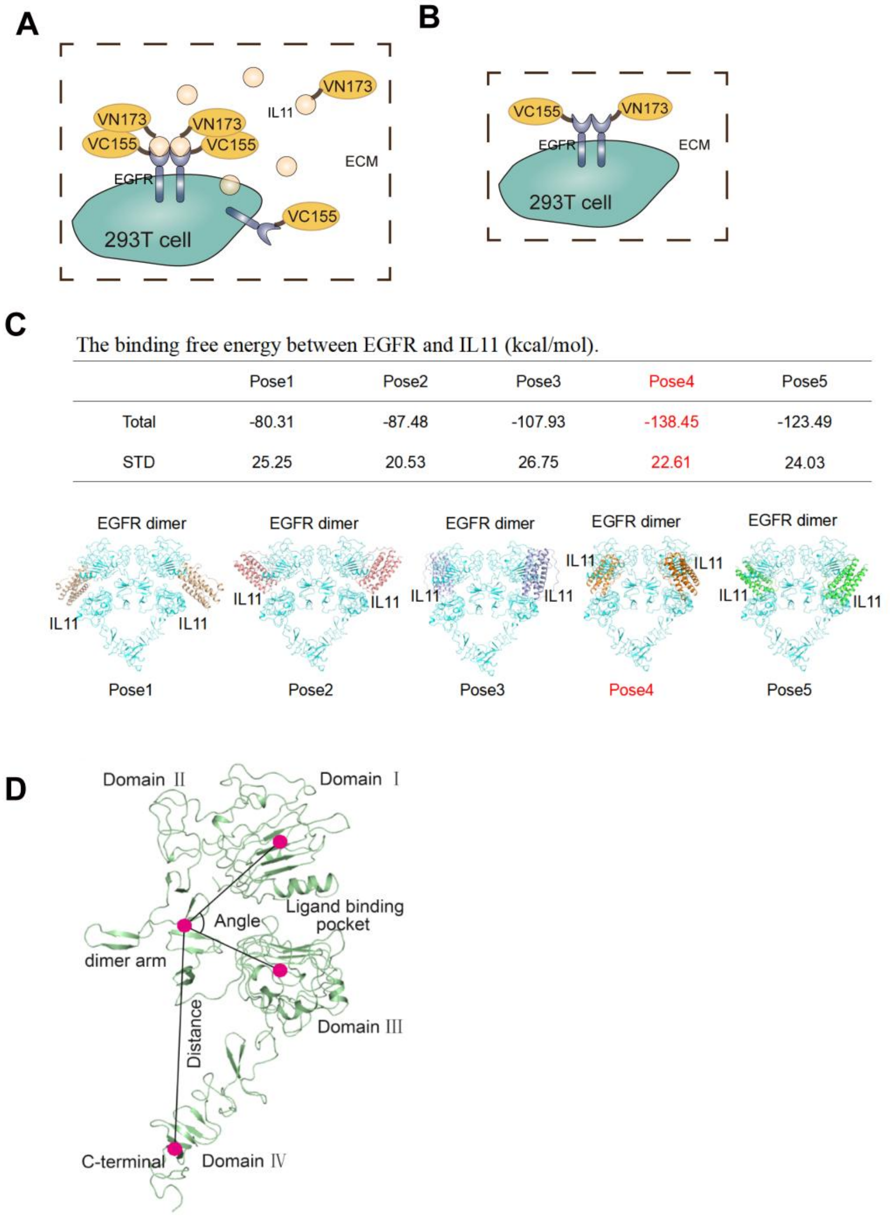

The binding free energy between EGFR and IL11 (kcal/mol).

	Pose1	Pose2	Pose3	Pose4	Pose5
Total	-80.31	-87.48	-107.93	-138.45	-123.49
STD	25.25	20.53	26.75	22.61	24.03

Figure S5 IL11 binds to EGFR as a ligand and stimulates EGFR phosphorylation.

(A-B) EGFR and IL11 constructs fused with C-terminal and N-terminal Venus halves (VC155) and (VN173) were expressed in human 293T cell lines. EGFR dimer formed by EGFR-VC155 and EGFR-VN173 were expressed in human 293T cell lines acted as positive control. **(C)** The five binding poses of EGFR and IL11 obtained by molecular docking and the binding free energy between EGFR and IL11 for the five binding poses. **(D)** The angle between centroids of domain I, part of domain II, and centroids of domain III was used to

estimate the degree of closure of the ligand-binding site of EGFR. The distance between part of domain II and C-terminal of domain IV was used to characterize distance between the C-terminal of domain Ⅳ and the other three extracellular domains.

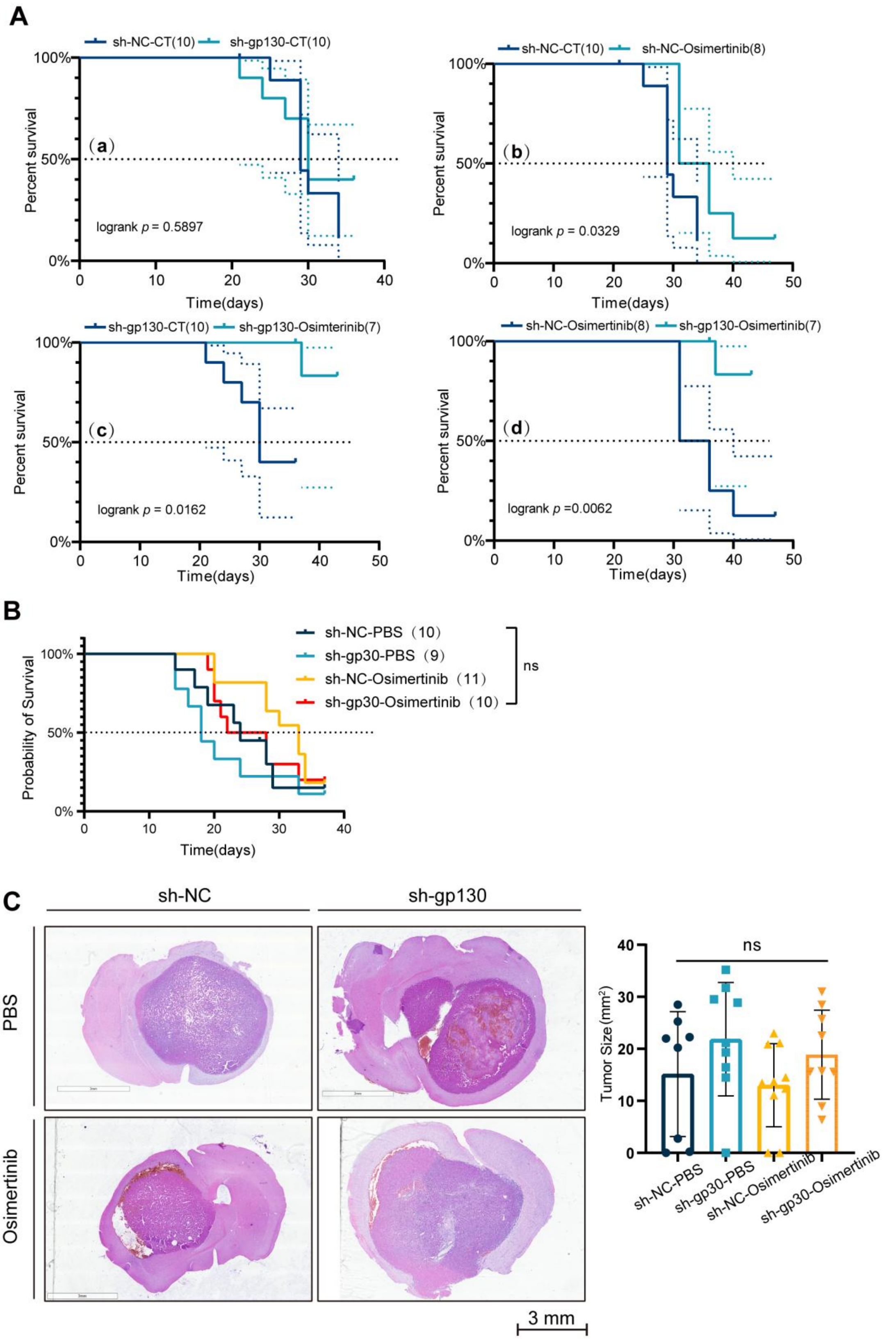

Figure S6 Combined targeting gp130 and EGFR inhibits PDL1 expression and restores T cell infiltration of BM.

(A) Survival curves of nude mice in each treatment groups (NC+PBS, n = 10; NC+OSI, n = 8; sh-gp130+PBS, n = 10; sh-gp130+OSI, n = 7; Log-rank (Mantel-Cox) test). **(B)** Survival curves of C57/6N mice in each treatment groups (NC+PBS, n = 10; sh-gp130+PBS, n = 9; NC+osimertinib, n = 11; sh-gp130+osimertinib, n = 10; Log-rank (Mantel-Cox) test). **(C)** Representative images of H&E staining. Tumor sizes of C57 mice in each treatment groups (NC+PBS, n = 8; sh-gp130+PBS, n = 9; NC+osimertinib, n = 10; sh-gp130+ osimertinib, n = 9; Unpaired, two-tailed *t*-test).

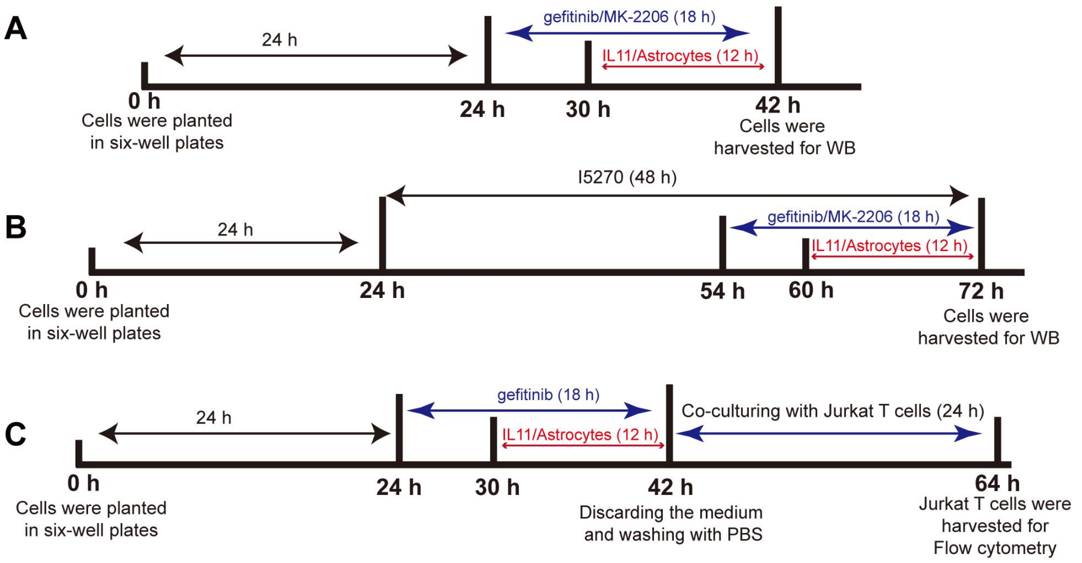

Figure S7 Three schematics of drugs and cells preconditioning for western blotting and flow cytometry.

(A, B) Two schematics of drugs and cells preconditioning for western blotting; **(C)** A schematic of drugs and cells preconditioning for flow cytometry.

Table S1 Correlation between CD3 infiltration and clinicopathological characteristics in patients with lung cancer.

Clinicopathological parameter	Variables	IL11 expression		total	χ2	*p* value
		Low	High			
Age					2.155	0.174
	< 60	13	7	20		
	≥ 60	17	21	38		
Gender					0.770	0.427
	Male	17	19	36		
	Female	13	9	22		
Histology					0.392	0.611
	Adenocarcinoma	27	25	52		
	Squamous	1	2	3		
	null	2	1	3		
T stage					3.150	0.138
	T1/T2	17	23	40		
	T3/T4	11	5	16		
	null	2	0	2		
N stage					5.784	***0.020***
	N0	12	20	32		
	N1/N2/N3	18	8	26		
M stage					36.460	***< 0.001***
	M0	3	25	28		
	M1	27	3	30		
TNM stage					25.226	***< 0.001***
	Ⅰ/Ⅱ	3	21	24		
	Ⅲ/Ⅳ	27	7	34		

Table S2 Correlation between CD8α infiltration and clinicopathological characteristics in patients with lung cancer.

Clinicopathological parameters	Variables	CD8α infiltration		Total	χ2	*P* value
		Low	High			
**Age**					0.141	0.777
	< 60	9	8	17		
	≥ 60	19	21	40		
**Gender**					0.856	0.417
	Male	16	20	36		
	Female	12	9	21		
**Histology**					0.220	1.000
	Adenocarcinoma	26	25	51		
	Squamous	2	3	5		
	Null	0	1	1		
**T stage**					6.437	***0.017***
	T1/T2	15	25	40		
	T3/T4	12	4	16		
	Null	1	0	1		
**N stage**					8.743	***0.007***
	N0	7	18	25		
	N1/N2/N3	21	10	31		
	Null	0	1	1		
**M stage**					19.097	***< 0.001***
	M0	6	23	29		
	M1	22	6	28		
**TNM stage**					11.246	***0.001***
	Ⅰ/Ⅱ	6	19	25		
	Ⅲ/Ⅳ	22	10	32		

Table S3 Gene list up- or down-regulated in astrocytes co-cultured with PC9-BrM3 (padj ≤ 10^{-10})

	number	gene_id	log2FoldChange	padj	gene_name
up	1	ENSG00000176171	2.198714035	2.93E-55	BNIP3
	2	ENSG00000111674	2.363146622	8.52E-43	ENO2
	3	ENSG00000162433	1.828135638	5.23E-37	AK4
	4	ENSG00000159399	1.604549692	1.96E-34	HK2
	5	ENSG00000183864	1.954377558	9.44E-31	TOB2
	6	ENSG00000034510	1.669470287	4.83E-29	TMSB10
	7	ENSG00000214193	2.376814672	1.37E-27	SH3D21
	8	ENSG00000114268	1.859025556	1.65E-27	PFKFB4
	9	ENSG00000247095	3.184932262	8.02E-27	MIR210HG
	10	ENSG00000107223	1.80790861	2.74E-24	EDF1
	11	ENSG00000228474	1.747486868	2.26E-23	OST4
	12	ENSG00000168209	3.088371619	2.05E-22	DDIT4
	13	ENSG00000112715	1.576127809	7.50E-21	VEGFA
	14	ENSG00000152256	1.620601949	1.65E-20	PDK1
	15	ENSG00000170525	1.129923451	3.01E-19	PFKFB3
	16	ENSG00000117394	1.475638336	8.60E-19	SLC2A1
	17	ENSG00000166337	2.06717051	1.82E-18	TAF10
	18	ENSG00000159388	1.72437944	2.11E-18	BTG2
	19	ENSG00000213859	1.69264171	7.83E-18	KCTD11
	20	ENSG00000163516	1.343302915	2.72E-17	ANKZF1
	21	ENSG00000204389	1.279990225	1.66E-16	HSPA1A
	22	ENSG00000167772	1.64831383	4.13E-16	ANGPTL4
	23	ENSG00000130332	1.863955699	1.40E-15	LSM7
	24	ENSG00000178882	1.16061794	1.41E-15	RFLNA
	25	ENSG00000204220	2.214100864	1.51E-15	PFDN6
	26	ENSG00000115461	0.95850466	2.70E-15	IGFBP5
	27	ENSG00000179772	1.734540024	4.45E-15	FOXS1
	28	ENSG00000135766	1.10219139	5.25E-15	EGLN1
	29	ENSG00000134333	0.912708177	5.68E-15	LDHA
	30	ENSG00000143847	5.580639937	9.21E-15	PPFIA4
	31	ENSG00000188483	2.263890467	9.81E-15	IER5L
	32	ENSG00000135245	1.662468942	2.14E-14	HILPDA
	33	ENSG00000145912	1.753764683	2.35E-14	NHP2
	34	ENSG00000100311	1.847212471	2.47E-14	PDGFB
	35	ENSG00000177700	2.002222154	6.04E-14	POLR2L
	36	ENSG00000113739	1.21243851	6.85E-14	STC2

37	ENSG00000005238	0.908322621	8.00E-14	FAM214B
38	ENSG00000126001	1.087562802	1.59E-13	CEP250
39	ENSG00000169991	1.125121101	1.59E-13	IFFO2
40	ENSG00000111678	1.559987239	1.92E-13	C12orf57
41	ENSG00000205155	2.975956982	2.03E-13	PSENEN
42	ENSG00000104881	1.263078464	6.96E-13	PPP1R13L
43	ENSG00000107796	1.02477149	1.08E-12	ACTA2
44	ENSG00000185215	1.166294895	1.25E-12	TNFAIP2
45	ENSG00000146094	2.078123764	1.31E-12	DOK3
46	ENSG00000188070	0.991639909	1.32E-12	C11orf95
47	ENSG00000241468	1.544232268	2.33E-12	ATP5MF
48	ENSG00000143631	1.028091071	3.95E-12	FLG
49	ENSG00000177106	1.337775314	4.97E-12	EPS8L2
50	ENSG00000186918	1.729970189	5.22E-12	ZNF395
51	ENSG00000170379	2.407640087	6.71E-12	TCAF2
52	ENSG00000117318	0.882567441	7.07E-12	ID3
53	ENSG00000232480	2.492737858	8.19E-12	TGFB2-AS1
54	ENSG00000095752	1.385655227	8.62E-12	IL11
55	ENSG00000104812	0.908140538	1.24E-11	GYS1
56	ENSG00000177469	0.856844388	2.11E-11	CAVIN1
57	ENSG00000124733	1.18491659	2.20E-11	MEA1
58	ENSG00000099849	1.98127732	2.23E-11	RASSF7
59	ENSG00000114023	1.224847393	4.24E-11	FAM162A
60	ENSG00000254726	1.330174513	4.26E-11	MEX3A
61	ENSG00000129521	1.807955809	8.97E-11	EGLN3
62	ENSG00000198467	1.588225249	1.06E-10	TPM2
63	ENSG00000188549	1.102116744	1.25E-10	CCDC9B
64	ENSG00000064666	0.901119551	1.26E-10	CNN2
65	ENSG00000069399	1.299304662	1.42E-10	BCL3
66	ENSG00000108852	1.61881158	1.74E-10	MPP2
67	ENSG00000143314	1.636186755	1.90E-10	MRPL24
68	ENSG00000067057	0.795866449	2.13E-10	PFKP
69	ENSG00000166924	3.072209628	2.46E-10	NYAP1
70	ENSG00000159167	1.409227996	2.88E-10	STC1
71	ENSG00000161016	1.364732708	3.79E-10	RPL8
72	ENSG00000065978	1.105123059	3.88E-10	YBX1
73	ENSG00000187840	1.14758147	4.01E-10	EIF4EBP1
74	ENSG00000102870	0.903991384	7.02E-10	ZNF629
75	ENSG00000141582	1.211126162	7.55E-10	CBX4

	76	ENSG00000176845	1.103672003	7.55E-10	METRNL
	77	ENSG00000145901	0.895095877	7.79E-10	TNIP1
	78	ENSG00000100142	1.237451688	7.80E-10	POLR2F
down	1	ENSG00000178695	-0.998422015	2.96E-13	KCTD12
	2	ENSG00000171522	-1.170294711	7.09E-11	PTGER4
	3	ENSG00000122862	-1.532880977	2.07E-10	SRGN
	4	ENSG00000081320	-0.843863992	5.76E-10	STK17B
	5	ENSG00000168542	-0.663925403	7.80E-10	COL3A1
	6	ENSG00000156273	-1.01001616	8.19E-10	BACH1

Table S4 Correlation between IL11 expression and clinicopathological characteristics in patients with lung cancer.

Clinicopathological parameters	Variables	IL11 expression		Total	χ2	*P* value
		Low	High			
**Age**					0.156	0.782
	< 60	10	8	18		
	≥ 60	21	21	42		
**Gender**					1.602	0.292
	Male	21	15	36		
	Female	10	14	24		
**Histology**					0.150	1.000
	Adenocarcinoma	27	26	53		
	Squamous	3	2	5		
	Null	1	1	2		
**T stage**					4.8	***0.040***
	T1/T2	26	15	41		
	T3/T4	5	11	16		
	Null	0	3	3		
**N stage**					2.379	0.196
	N0	19	12	31		
	N1/N2/N3	12	17	29		
**M stage**					24.093	**< *0.001***
	M0	25	5	30		
	M1	6	24	30		
**TNM stage**					15.561	**< *0.001***
	Ⅰ/Ⅱ	21	5	26		
	Ⅲ/Ⅳ	10	24	34		

Table S5 Correlation analysis of IL11 expression and clinicopathological imaging features in serum validated cohort of lung cancer patients with brain metastases.

Characteristics	Case	IL11_*P*
**Gender**		0.790
Male	13 (54.1%)	
Female	11 (45.9%)	
**Age**		0.265
≤ 60	13 (54.1%)	
> 60	11 (45.9%)	
**Pathological type**		—
Adenocarcinoma	23 (95.8%)	
Squamous	1 (4.2%)	
**Smoke**		0.106
With	7 (29.2%)	
Without	17 (70.8%)	
**Other organ metastasis**		***0.022***
With	17 (70.8%)	
Without	7 (29.2%)	
**Number of brain metastatic lesions**		0.551
1	10 (41.7%)	
≥ 2	14 (58.3%)	
**Maximum diameter of BM (cm)**		0.340
≤ 2	22 (91.7%)	
> 2	2 (8.3%)	
**Meningeal metastasis**		0.858
With	4 (16.7%)	
Without	20 (83.3%)	
**T stage**		0.698
T1	3 (12.5%)	
T2	5 (20.9%)	
T3	6 (25%)	
T4	8 (33.3%)	
Tx	2 (8.3%)	
**N stage**		0.219
N0	0	
N1	0	
N2	12 (50%)	
N3	10 (41.7%)	
Nx	2 (8.3%)	

Table S6 Clinicopathologic imaging features of the serum validated cohort.

Characteristics	Case	Characteristics	Case
**Lung cancer brain metastasis**	24	**Controls**	74
**Gender**		**Gender**	
Male	13	Male	29
Female	11	Female	45
**Age**		**Age**	
≤ 60	13	≤ 60	27
> 60	11	> 60	47
**Pathological type**		**Type**	
Adenocarcinoma	23	NSCLC	63
Squamous	1	Healthy group	11
**Smoke**		**Pathological type**	
With	7	Adenocarcinoma	53
Without	17	Squamous	8
**Other organ metastasis**		Other types	2
With	17	**TNM stage**	
Without	7	Ⅰ/Ⅱ	24
**Number of brain metastatic lesions**		Ⅲ/Ⅳ	39
1	10	**M stage**	
≥ 2	14	M0	27
**Maximum diameter of BM (cm)**		M1	36
≤ 2	22	**T stage**	
> 2	2	T1	22
**Meningeal metastasis**		T2	8
With	4	T3	12
Without	20	T4	20
**T stage**		Null	1
T1	3	**N stage**	
T2	5	N0	28
T3	6	N1	1
T4	8	N2	15
Tx	2	N3	19
**N stage**			
N0	0		
N1	0		
N2	12		
N3	10		
Nx	2		

Table S7 Clinicopathologic features of the CSF validated cohort.

Characteristics	Case	Characteristics	Case
**Lung cancer brain metastasis**	7	**Controls**	6
**Gender**		**Gender**	
Male	3	Male	3
Female	4	Female	3
**Age**		**Age**	
≤ 60	3	≤ 60	4
> 60	4	> 60	2
**Other organ metastasis**		**Diagnosis**	
With	6	Lymphoma	4
Without	1	Lacunar infarction	1
		Meningioma	1

Table S8 Dominant residues in binding free energy contributions between EGFR and IL11 (kcal/mol)

Protein	Residue	Total	STD
EGFR	F381	-5.53	1.16
	L38	-4.68	1.12
	E114	-3.90	1.17
	R149	-3.29	0.74
	T382	-2.02	1.32
	D179	-2.82	1.31
	N152	-2.74	1.21
	S123	-1.95	0.52
	K346	-1.84	0.40
	N115	-1.44	0.37
IL11	R98	-7.84	0.41
	R138	-4.11	1.30
	Q151	-2.75	0.95
	L142	-2.65	0.37
	P154	-1.91	0.29
	L102	-1.90	0.11
	D155	-1.87	0.45
	P150	-1.80	0.25
	D134	-1.51	0.21
	R135	-1.28	0.42

Table S9 The sequences for fusion genes of C-terminal (VC155) with EGFR, N-terminal (VN173) with IL11 and VN173 with EGFR

NAME	Sequence
EGFR-VC155	5’-ATGCGACCCTCCGGGACGGCCGGGGCAGCGCTCCTGGCGCTGCTGG CTGCGCTCTGCCCGGCGAGTCGGGCTCTGGAGGAAAAGAAAGTTTGCC AAGGCACGAGTAACAAGCTCACGCAGTTGGGCACTTTTGAAGATCATT TTCTCAGCCTCCAGAGGATGTTCAATAACTGTGAGGTGGTCCTTGGGAA TTTGGAAATTACCTATGTGCAGAGGAATTATGATCTTTCCTTCTTAAAG ACCATCCAGGAGGTGGCTGGTTATGTCCTCATTGCCCTCAACACAGTGG AGCGAATTCCTTTGGAAAACCTGCAGATCATCAGAGGAAATATGTACT ACGAAAATTCCTATGCCTTAGCAGTCTTATCTAACTATGATGCAAATAA AACCGGACTGAAGGAGCTGCCCATGAGAAATTTACAGGAAATCCTGCA TGGCGCCGTGCGGTTCAGCAACAACCCTGCCCTGTGCAACGTGGAGAG CATCCAGTGGCGGGACATAGTCAGCAGTGACTTTCTCAGCAACATGTC GATGGACTTCCAGAACCACCTGGGCAGCTGCCAAAAGTGTGATCCAAG CTGTCCCAATGGGAGCTGCTGGGGTGCAGGAGAGGAGAACTGCCAGAA ACTGACCAAAATCATCTGTGCCCAGCAGTGCTCCGGGCGCTGCCGTGG CAAGTCCCCCAGTGACTGCTGCCACAACCAGTGTGCTGCAGGCTGCAC AGGCCCCCGGGAGAGCGACTGCCTGGTCTGCCGCAAATTCCGAGACGA AGCCACGTGCAAGGACACCTGCCCCCCACTCATGCTCTACAACCCCACC ACGTACCAGATGGATGTGAACCCCGAGGGCAAATACAGCTTTGGTGCC ACCTGCGTGAAGAAGTGTCCCCGTAATTATGTGGTGACAGATCACGGC TCGTGCGTCCGAGCCTGTGGGGCCGACAGCTATGAGATGGAGGAAGAC GGCGTCCGCAAGTGTAAGAAGTGCGAAGGGCCTTGCCGCAAAGTGTGT AACGGAATAGGTATTGGTGAATTTAAAGACTCACTCTCCATAAATGCTA CGAATATTAAACACTTCAAAAACTGCACCTCCATCAGTGGCGATCTCCA CATCCTGCCGGTGGCATTTAGGGGTGACTCCTTCACACATACTCCTCCT CTGGATCCACAGGAACTGGATATTCTGAAAACCGTAAAGGAAATCACA GGGTTTTTGCTGATTCAGGCTTGGCCTGAAAACAGGACGGACCTCCATG CCTTTGAGAACCTAGAAATCATACGCGGCAGGACCAAGCAACATGGTC AGTTTTCTCTTGCAGTCGTCAGCCTGAACATAACATCCTTGGGATTACG CTCCCTCAAGGAGATAAGTGATGGAGATGTGATAATTTCAGGAAACAA AAATTTGTGCTATGCAAATACAATAAACTGGAAAAAACTGTTTGGGAC CTCCGGTCAGAAAACCAAAATTATAAGCAACAGAGGTGAAAACAGCTG CAAGGCCACAGGCCAGGTCTGCCATGCCTTGTGCTCCCCCGAGGGCTG CTGGGGCCCGGAGCCCAGGGACTGCGTCTCTTGCCGGAATGTCAGCCG AGGCAGGGAATGCGTGGACAAGTGCAACCTTCTGGAGGGTGAGCCAAG GGAGTTTGTGGAGAACTCTGAGTGCATACAGTGCCACCCAGAGTGCCT GCCTCAGGCCATGAACATCACCTGCACAGGACGGGGACCAGACAACTG TATCCAGTGTGCCCACTACATTGACGGCCCCCACTGCGTCAAGACCTGC CCGGCAGGAGTCATGGGAGAAAACAACACCCTGGTCTGGAAGTACGCA GACGCCGGCCATGTGTGCCACCTGTGCCATCCAAACTGCACCTACGGAT

	GCACTGGGCCAGGTCTTGAAGGCTGTCCAACGAATGGGCCTAAGATCC<br>CGTCCATCGCCACTGGGATGGTGGGGGCCCTCCTCTTGCTGCTGGTGGT<br>GGCCCTGGGGATCGGCCTCTTCATGCGAAGGCGCCACATCGTTCGGAA<br>GCGCACGCTGCGGAGGCTGCTGCAGGAGAGGGAGCTTGTGGAGCCTCT<br>TACACCCAGTGGAGAAGCTCCCAACCAAGCTCTCTTGAGGATCTTGAA<br>GGAAACTGAATTCAAAAAGATCAAAGTGCTGGGCTCCGGTGCGTTCGG<br>CACGGTGTATAAGGGACTCTGGATCCCAGAAGGTGAGAAAGTTAAAAT<br>TCCCGTCGCTATCAAGGAATTAAGAGAAGCAACATCTCCGAAAGCCAA<br>CAAGGAAATCCTCGATGAAGCCTACGTGATGGCCAGCGTGGACAACCC<br>CCACGTGTGCCGCCTGCTGGGCATCTGCCTCACCTCCACCGTGCAGCTC<br>ATCACGCAGCTCATGCCCTTCGGCTGCCTCCTGGACTATGTCCGGGAAC<br>ACAAAGACAATATTGGCTCCCAGTACCTGCTCAACTGGTGTGTGCAGAT<br>CGCAAAGGGCATGAACTACTTGGAGGACCGTCGCTTGGTGCACCGCGA<br>CCTGGCAGCCAGGAACGTACTGGTGAAAACACCGCAGCATGTCAAGAT<br>CACAGATTTTGGGCTGGCCAAACTGCTGGGTGCGGAAGAGAAAGAATA<br>CCATGCAGAAGGAGGCAAAGTGCCTATCAAGTGGATGGCATTGGAATC<br>AATTTTACACAGAATCTATACCCACCAGAGTGATGTCTGGAGCTACGG<br>GGTGACTGTTTGGGAGTTGATGACCTTTGGATCCAAGCCATATGACGGA<br>ATCCCTGCCAGCGAGATCTCCTCCATCCTGGAGAAAGGAGAACGCCTC<br>CCTCAGCCACCCATATGTACCATCGATGTCTACATGATCATGGTCAAGT<br>GCTGGATGATAGACGCAGATAGTCGCCCAAAGTTCCGTGAGTTGATCA<br>TCGAATTCTCCAAAATGGCCCGAGACCCCCAGCGCTACCTTGTCATTCA<br>GGGGGATGAAAGAATGCATTTGCCAAGTCCTACAGACTCCAACTTCTA<br>CCGTGCCCTGATGGATGAAGAAGACATGGACGACGTGGTGGATGCCGA<br>CGAGTACCTCATCCCACAGCAGGGCTTCTTCAGCAGCCCCTCCACGTCA<br>CGGACTCCCCTCCTGAGCTCTCTGAGTGCAACCAGCAACAATTCCACCG<br>TGGCTTGCATTGATAGAAATGGGCTGCAAAGCTGTCCCATCAAGGAAG<br>ACAGCTTCTTGCAGCGATACAGCTCAGACCCCACAGGCGCCTTGACTG<br>AGGACAGCATAGACGACACCTTCCTCCCAGTGCCTGAATACATAAACC<br>AGTCCGTTCCCAAAAGGCCCGCTGGCTCTGTGCAGAATCCTGTCTATCA<br>CAATCAGCCTCTGAACCCCGCGCCCAGCAGAGACCCACACTACCAGGA<br>CCCCCACAGCACTGCAGTGGGCAACCCCGAGTATCTCAACACTGTCCA<br>GCCCACCTGTGTCAACAGCACATTCGACAGCCCTGCCCACTGGGCCCA<br>GAAAGGCAGCCACCAAATTAGCCTGGACAACCCTGACTACCAGCAGGA<br>CTTCTTTCCCAAGGAAGCCAAGCCAAATGGCATCTTTAAGGGCTCCACA<br>GCTGAAAATGCAGAATACCTAAGGGTCGCGCCACAAAGCAGTGAATTT<br>ATTGGAGCAGGAGGTGGAGGATCAATGAACCACGACAAGCAGAAGAA<br>CGGCATCAAGGCCAACTTCAAGATCCGCCACAACATCGAGGACGGCGG<br>CGTGCAGCTCGCCGACCACTACCAGCAGAACACCCCCATCGGCGACGG<br>CCCCGTGCTGCTGCCCGACAACCACTACCTGAGCTACCAGTCCAAACTG<br>AGCAAAGACCCCAACGAGAAGCGCGATCACATGGTCCTGCTGGAGTTC<br>GTGACCGCCGCCGGGATCACTCTCGGCATGGACGAGCTGTACAAGTAA-<br>3’

IL11-VN173	5’-ATGAACTGTGTTTGCCGCCTGGTCCTGGTCGTGCTGAGCCTGTGGCC<br>AGATACAGCTGTCGCCCCTGGGCCACCACCTGGCCCCCCTCGAGTTTCC<br>CCAGACCCTCGGGCCGAGCTGGACAGCACCGTGCTCCTGACCCGCTCTC<br>TCCTGGCGGACACGCGGCAGCTGGCTGCACAGCTGAGGGACAAATTCC<br>CAGCTGACGGGGACCACAACCTGGATTCCCTGCCCACCCTGGCCATGA<br>GTGCGGGGGCACTGGGAGCTCTACAGCTCCCAGGTGTGCTGACAAGGC<br>TGCGAGCGGACCTACTGTCCTACCTGCGGCACGTGCAGTGGCTGCGCC<br>GGGCAGGTGGCTCTTCCCTGAAGACCCTGGAGCCCGAGCTGGGCACCC<br>TGCAGGCCCGACTGGACCGGCTGCTGCGCCGGCTGCAGCTCCTGATGTC<br>CCGCCTGGCCCTGCCCCAGCCACCCCCGGACCCGCCGGCGCCCCCGCTG<br>GCGCCCCCCTCCTCAGCCTGGGGGGGCATCAGGGCCGCCCACGCCATC<br>CTGGGGGGGCTGCACCTGACACTTGACTGGGCCGTGAGGGGACTGCTG<br>CTGCTGAAGACTCGGCTGGGAGGTGGAGGATCAATGGTGAGCAAGGGC<br>GAGGAGCTGTTCACCGGGGTGGTGCCCATCCTGGTCGAGCTGGACGGC<br>GACGTAAACGGCCACAAGTTCAGCGTGTCCGGCGAGGGCGAGGGCGAT<br>GCCACCTACGGCAAGCTGACCCTGAAGCTGATCTGCACCACCGGCAAG<br>CTGCCCGTGCCCTGGCCCACCCTCGTGACCACCCTGGGCTACGGCCTGC<br>AGTGCTTCGCCCGCTACCCCGACCACATGAAGCAGCACGACTTCTTCAA<br>GTCCGCCATGCCCGAAGGCTACGTCCAGGAGCGCACCATCTTCTTCAAG<br>GACGACGGCAACTACAAGACCCGCGCCGAGGTGAAGTTCGAGGGCGA<br>CACCCTGGTGAACCGCATCGAGCTGAAGGGCATCGACTTCAAGGAGGA<br>CGGCAACATCCTGGGGCACAAGCTGGAGTACAACTACAACAGCCACAA<br>CGTCTATATCACCGCCGACAAGCAGAAGAACGGCATCAAGGCCAACTT<br>CAAGATCCGCCACAACATCGAGTAG-3’
EGFR-VN173	5’-ATGCGACCCTCCGGGACGGCCGGGGCAGCGCTCCTGGCGCTGCTGG<br>CTGCGCTCTGCCCGGCGAGTCGGGCTCTGGAGGAAAAGAAAGTTTGCC<br>AAGGCACGAGTAACAAGCTCACGCAGTTGGGCACTTTTGAAGATCATT<br>TTCTCAGCCTCCAGAGGATGTTCAATAACTGTGAGGTGGTCCTTGGGAA<br>TTTGGAAATTACCTATGTGCAGAGGAATTATGATCTTTCCTTCTTAAAG<br>ACCATCCAGGAGGTGGCTGGTTATGTCCTCATTGCCCTCAACACAGTGG<br>AGCGAATTCCTTTGGAAAACCTGCAGATCATCAGAGGAAATATGTACT<br>ACGAAAATTCCTATGCCTTAGCAGTCTTATCTAACTATGATGCAAATAA<br>AACCGGACTGAAGGAGCTGCCCATGAGAAATTTACAGGAAATCCTGCA<br>TGGCGCCGTGCGGTTCAGCAACAACCCTGCCCTGTGCAACGTGGAGAG<br>CATCCAGTGGCGGGACATAGTCAGCAGTGACTTTCTCAGCAACATGTC<br>GATGGACTTCCAGAACCACCTGGGCAGCTGCCAAAAGTGTGATCCAAG<br>CTGTCCCAATGGGAGCTGCTGGGGTGCAGGAGAGGAGAACTGCCAGAA<br>ACTGACCAAAATCATCTGTGCCCAGCAGTGCTCCGGGCGCTGCCGTGG<br>CAAGTCCCCCAGTGACTGCTGCCACAACCAGTGTGCTGCAGGCTGCAC<br>AGGCCCCCGGGAGAGCGACTGCCTGGTCTGCCGCAAATTCCGAGACGA<br>AGCCACGTGCAAGGACACCTGCCCCCCACTCATGCTCTACAACCCCACC<br>ACGTACCAGATGGATGTGAACCCCGAGGGCAAATACAGCTTTGGTGCC<br>ACCTGCGTGAAGAAGTGTCCCCGTAATTATGTGGTGACAGATCACGGC

	TCGTGCGTCCGAGCCTGTGGGGCCGACAGCTATGAGATGGAGGAAGAC<br>GGCGTCCGCAAGTGTAAGAAGTGCGAAGGGCCTTGCCGCAAAGTGTGT<br>AACGGAATAGGTATTGGTGAATTTAAAGACTCACTCTCCATAAATGCTA<br>CGAATATTAAACACTTCAAAAACTGCACCTCCATCAGTGGCGATCTCCA<br>CATCCTGCCGGTGGCATTTAGGGGTGACTCCTTCACACATACTCCTCCT<br>CTGGATCCACAGGAACTGGATATTCTGAAAACCGTAAAGGAAATCACA<br>GGGTTTTTGCTGATTCAGGCTTGGCCTGAAAACAGGACGGACCTCCATG<br>CCTTTGAGAACCTAGAAATCATACGCGGCAGGACCAAGCAACATGGTC<br>AGTTTTCTCTTGCAGTCGTCAGCCTGAACATAACATCCTTGGGATTACG<br>CTCCCTCAAGGAGATAAGTGATGGAGATGTGATAATTTCAGGAAACAA<br>AAATTTGTGCTATGCAAATACAATAAACTGGAAAAAACTGTTTGGGAC<br>CTCCGGTCAGAAAACCAAAATTATAAGCAACAGAGGTGAAAACAGCTG<br>CAAGGCCACAGGCCAGGTCTGCCATGCCTTGTGCTCCCCCGAGGGCTG<br>CTGGGGCCCGGAGCCCAGGGACTGCGTCTCTTGCCGGAATGTCAGCCG<br>AGGCAGGGAATGCGTGGACAAGTGCAACCTTCTGGAGGGTGAGCCAAG<br>GGAGTTTGTGGAGAACTCTGAGTGCATACAGTGCCACCCAGAGTGCCT<br>GCCTCAGGCCATGAACATCACCTGCACAGGACGGGGACCAGACAACTG<br>TATCCAGTGTGCCCACTACATTGACGGCCCCCACTGCGTCAAGACCTGC<br>CCGGCAGGAGTCATGGGAGAAAACAACACCCTGGTCTGGAAGTACGCA<br>GACGCCGGCCATGTGTGCCACCTGTGCCATCCAAACTGCACCTACGGAT<br>GCACTGGGCCAGGTCTTGAAGGCTGTCCAACGAATGGGCCTAAGATCC<br>CGTCCATCGCCACTGGGATGGTGGGGGCCCTCCTCTTGCTGCTGGTGGT<br>GGCCCTGGGGATCGGCCTCTTCATGCGAAGGCGCCACATCGTTCGGAA<br>GCGCACGCTGCGGAGGCTGCTGCAGGAGAGGGAGCTTGTGGAGCCTCT<br>TACACCCAGTGGAGAAGCTCCCAACCAAGCTCTCTTGAGGATCTTGAA<br>GGAAACTGAATTCAAAAAGATCAAAGTGCTGGGCTCCGGTGCGTTCGG<br>CACGGTGTATAAGGGACTCTGGATCCCAGAAGGTGAGAAAGTTAAAAT<br>TCCCGTCGCTATCAAGGAATTAAGAGAAGCAACATCTCCGAAAGCCAA<br>CAAGGAAATCCTCGATGAAGCCTACGTGATGGCCAGCGTGGACAACCC<br>CCACGTGTGCCGCCTGCTGGGCATCTGCCTCACCTCCACCGTGCAGCTC<br>ATCACGCAGCTCATGCCCTTCGGCTGCCTCCTGGACTATGTCCGGGAAC<br>ACAAAGACAATATTGGCTCCCAGTACCTGCTCAACTGGTGTGTGCAGAT<br>CGCAAAGGGCATGAACTACTTGGAGGACCGTCGCTTGGTGCACCGCGA<br>CCTGGCAGCCAGGAACGTACTGGTGAAAACACCGCAGCATGTCAAGAT<br>CACAGATTTTGGGCTGGCCAAACTGCTGGGTGCGGAAGAGAAAGAATA<br>CCATGCAGAAGGAGGCAAAGTGCCTATCAAGTGGATGGCATTGGAATC<br>AATTTTACACAGAATCTATACCCACCAGAGTGATGTCTGGAGCTACGG<br>GGTGACTGTTTGGGAGTTGATGACCTTTGGATCCAAGCCATATGACGGA<br>ATCCCTGCCAGCGAGATCTCCTCCATCCTGGAGAAAGGAGAACGCCTC<br>CCTCAGCCACCCATATGTACCATCGATGTCTACATGATCATGGTCAAGT<br>GCTGGATGATAGACGCAGATAGTCGCCCAAAGTTCCGTGAGTTGATCA<br>TCGAATTCTCCAAAATGGCCCGAGACCCCCAGCGCTACCTTGTCATTCA<br>GGGGGATGAAAGAATGCATTTGCCAAGTCCTACAGACTCCAACTTCTA<br>CCGTGCCCTGATGGATGAAGAAGACATGGACGACGTGGTGGATGCCGA

	CGAGTACCTCATCCCACAGCAGGGCTTCTTCAGCAGCCCCTCCACGTCA<br>CGGACTCCCCTCCTGAGCTCTCTGAGTGCAACCAGCAACAATTCCACCG<br>TGGCTTGCATTGATAGAAATGGGCTGCAAAGCTGTCCCATCAAGGAAG<br>ACAGCTTCTTGCAGCGATACAGCTCAGACCCCACAGGCGCCTTGACTG<br>AGGACAGCATAGACGACACCTTCCTCCCAGTGCCTGAATACATAAACC<br>AGTCCGTTCCCAAAAGGCCCGCTGGCTCTGTGCAGAATCCTGTCTATCA<br>CAATCAGCCTCTGAACCCCGCGCCCAGCAGAGACCCACACTACCAGGA<br>CCCCCACAGCACTGCAGTGGGCAACCCCGAGTATCTCAACACTGTCCA<br>GCCCACCTGTGTCAACAGCACATTCGACAGCCCTGCCCACTGGGCCCA<br>GAAAGGCAGCCACCAAATTAGCCTGGACAACCCTGACTACCAGCAGGA<br>CTTCTTTCCCAAGGAAGCCAAGCCAAATGGCATCTTTAAGGGCTCCACA<br>GCTGAAAATGCAGAATACCTAAGGGTCGCGCCACAAAGCAGTGAATTT<br>ATTGGAGCAGGAGGTGGAGGATCAATGGTGAGCAAGGGCGAGGAGCT<br>GTTCACCGGGGTGGTGCCCATCCTGGTCGAGCTGGACGGCGACGTAAA<br>CGGCCACAAGTTCAGCGTGTCCGGCGAGGGCGAGGGCGATGCCACCTA<br>CGGCAAGCTGACCCTGAAGCTGATCTGCACCACCGGCAAGCTGCCCGT<br>GCCCTGGCCCACCCTCGTGACCACCCTGGGCTACGGCCTGCAGTGCTTC<br>GCCCGCTACCCCGACCACATGAAGCAGCACGACTTCTTCAAGTCCGCC<br>ATGCCCGAAGGCTACGTCCAGGAGCGCACCATCTTCTTCAAGGACGAC<br>GGCAACTACAAGACCCGCGCCGAGGTGAAGTTCGAGGGCGACACCCTG<br>GTGAACCGCATCGAGCTGAAGGGCATCGACTTCAAGGAGGACGGCAAC<br>ATCCTGGGGCACAAGCTGGAGTACAACTACAACAGCCACAACGTCTAT<br>ATCACCGCCGACAAGCAGAAGAACGGCATCAAGGCCAACTTCAAGATC<br>CGCCACAACATCGAGTAG-3’

ShRNA sequences for stable knockdown

NAME	Sequence
gp130 for Human	5’-GGUAAGGGAUACUGGAGUGTT-3’
gp130 for Mouse	5’-GCGTCTTGTTCTGCTTTAA-3’

Amino acid sequences of IL11 mutant protein

WT

PGPPPGPPRVSPDPRAELDSTVLLTRSLLADTRQLAAQLRDKFPADGDHNLDSLPTLA
MSAGALGALQLPGVLTRLRADLLSYLRHVQWLRRAGGSSLKTLEPELGTLQARLDR
LLRRLQLLMSRLALPQPPPDPPAPPLAPPSSAWGGIRAAHAILGGLHLTLDWAVRGLL
LLKTRL

R98D

PGPPPGPPRVSPDPRAELDSTVLLTRSLLADTRQLAAQLRDKFPADGDHNLDSLPTLA
MSAGALGALQLPGVLTRLDADLLSYLRHVQWLRRAGGSSLKTLEPELGTLQARLDR
LLRRLQLLMSRLALPQPPPDPPAPPLAPPSSAWGGIRAAHAILGGLHLTLDWAVRGLL
LLKTRL

R138D

PGPPPGPPRVSPDPRAELDSTVLLTRSLLADTRQLAAQLRDKFPADGDHNLDSLPTLA
MSAGALGALQLPGVLTRLRADLLSYLRHVQWLRRAGGSSLKTLEPELGTLQARLDR
LLDRLQLLMSRLALPQPPPDPPAPPLAPPSSAWGGIRAAHAILGGLHLTLDWAVRGLL
LLKTRL

Q151G

PGPPPGPPRVSPDPRAELDSTVLLTRSLLADTRQLAAQLRDKFPADGDHNLDSLPTLA
MSAGALGALQLPGVLTRLRADLLSYLRHVQWLRRAGGSSLKTLEPELGTLQARLDR
LLRRLQLLMSRLALPGPPPDPPAPPLAPPSSAWGGIRAAHAILGGLHLTLDWAVRGLL
LLKTRL

L142D

PGPPPGPPRVSPDPRAELDSTVLLTRSLLADTRQLAAQLRDKFPADGDHNLDSLPTLA
MSAGALGALQLPGVLTRLRADLLSYLRHVQWLRRAGGSSLKTLEPELGTLQARLDR
LLRRLQDLMSRLALPQPPPDPPAPPLAPPSSAWGGIRAAHAILGGLHLTLDWAVRGLL
LLKTRL

L102D

PGPPPGPPRVSPDPRAELDSTVLLTRSLLADTRQLAAQLRDKFPADGDHNLDSLPTLA
MSAGALGALQLPGVLTRLRADLDSYLRHVQWLRRAGGSSLKTLEPELGTLQARLDR
LLRRLQLLMSRLALPQPPPDPPAPPLAPPSSAWGGIRAAHAILGGLHLTLDWAVRGLL
LLKTRL
